# Insights into the therapeutic strategies for aging and aging-associated diseases

**DOI:** 10.1038/s41392-026-02662-z

**Published:** 2026-06-01

**Authors:** Ruifang Dong, Qiming Wu, Juntao Kan, Caili Fu, Vincenzo Sorrentino, Ashley Chow, Yanan Lei, Di Wu, Zhenzhen Xu, Jun Du, Dejian Huang

**Affiliations:** 1 National University of Singapore (Suzhou) Research Institute, Suzhou, China; 2 Healthy Aging Research Center, Nutrilite Health Institute, Shanghai, China; 3 Healthy Longevity Translational Research Programme, Yong Loo Lin School of Medicine, National University of Singapore, Singapore, Singapore; 4 Department of Biochemistry, Yong Loo Lin School of Medicine, National University of Singapore, Singapore, Singapore; 5 Institute of Quality Standards and Testing Technology for Agro-Products of Chinese Academy of Agricultural Sciences, Key Laboratory of Agro-food Safety and Quality, Ministry of Agriculture and Rural Affairs, Beijing, China; 6 Department of Food Science & Technology, National University of Singapore, Singapore, Singapore

**Keywords:** Drug screening, Drug development, Senescence, Epigenetics

## Abstract

Aging is a complex biological process characterized by progressive functional decline, driving the incidence of age-related diseases such as neurodegeneration, metabolic disorders, and cardiovascular diseases. Therapeutic strategies targeting aging hallmarks can delay aging and mitigate disease risk. Emerging interventions focus on modulating core aging mechanisms, including cellular senescence, metabolic dysfunction, epigenetic alterations, and mitochondrial impairment, etc. Recent advances have focused on three strategies: senolytics (eliminating senescent cells, e.g., dasatinib + quercetin), senomorphics (inhibiting the senescence-associated secretory phenotype, e.g., rapamycin), and senoreversion (rejuvenating senescent cells via epigenetic reprogramming). Additionally, metabolic interventions such as caloric restriction mimetics (e.g., spermidine, α-ketoglutarate, ergothioneine) enhance mitochondrial function, activate autophagy, and reprogram energy metabolism, demonstrating lifespan extension and healthspan improvement in preclinical models. Collectively, these approaches hold promise for delaying aging and alleviating age-related pathologies, facilitating the transition to precision longevity medicine. Concurrently, artificial intelligence (AI) accelerates discovery by integrating multiomics data, predicting candidate compounds, identifying biomarkers, and enabling personalized interventions. Despite advancements, challenges remain in target specificity, off-target effects, and clinical translation. The convergence of AI, multitarget strategies, and precision medicine signals a transformative era in extending healthspan and combating aging-associated diseases. This review systematically summarizes current breakthroughs, clinical landscapes, and future directions in aging therapeutics, underscoring interdisciplinary strategies to redefine healthy aging.

## Introduction

From a biological perspective, aging is the result of the gradual accumulation of various senescent cells and cell damage over time. This results in a gradual loss of physical and mental ability, an increased occurrence rate of illness and ultimately death. The global population aged 60 and above will double to 2.1 billion by 2050, and the population aged 80 and above will reach 426 million.^1^ China is one of the countries with the fastest aging rates in the world, and the proportion of people aged 60 and above is expected to reach 28% by 2040.^2^ This demographic transformation not only brings about social problems such as social security and the allocation of medical resources but also highlights the enormous pressure exerted by aging-related diseases (such as neurodegenerative disease, cardiovascular disease, diabetes, and sarcopenia) on the public health system. However, despite certain achievements in aging-related research, there are still many bottlenecks and gaps in the current understanding of aging mechanisms and the development of intervention methods. Hence, holistic molecular insights are urgently needed to help develop scientifically sound strategies, through foods, therapeutics, and lifestyle, to alleviate age-related diseases and achieve healthy aging.

Aging is a process of multidimensional homeostatic imbalance and functional degradation in tissues, organs, and cells. Its characteristics are the gradual decline of physiological integrity, resulting in functional impairment. Aging is a major risk factor for a variety of chronic diseases, including cancer, neurodegenerative disorders,^3,4^ cardiovascular diseases,^5^ metabolic syndromes (such as diabetes and obesity),^6^ and autoimmune diseases.^7^ While extensive research has identified key aging markers and explored the individual mechanisms underlying these diseases, several critical gaps remain: (1) the shared and disease-specific regulatory networks governing aging across different pathologies are poorly characterized; (2) the translation of mechanistic insights into effective therapeutic strategies is hindered by a lack of integration between basic research on aging mechanisms and the clinical exploration of interventions; and (3) existing aging-delaying approaches, such as rapamycin, senolytics, senomorphics, nutraceuticals and dietary interventions, have been studied in isolation with their long time safety and tissue specific impacts unclear. Furthermore, few studies have been reported on the synergistic effects of their combinations. These gaps highlight the need for an integrated framework to uncover the link between mechanistic understanding and therapeutic applications of these potential agents.

Herein, we attempt to clarify and construct a multidimensional research framework of “aging mechanisms, aging-related diseases, intervention measures, clinical applications”, aiming to strengthen the bridge between basic aging research and clinical translation. This review provides a comprehensive overview of the major markers of aging and the mechanisms regulating aging in various aging-related diseases, such as cancer, neurodegenerative diseases, cardiovascular diseases, metabolic diseases, autoimmune diseases and skin aging. We also discuss antiaging approaches, including geroprotectors, senolytics, senomorphics, senoreversion, natural molecules and dietary interventions. We expect to identify potential targets for these longevity interventions that reduce the risk of age-related diseases, delay the aging process and achieve healthy aging through the study of aging mechanisms. These interventions, ranging from pharmacological methods to dietary regulation, are expected to improve the quality of life of older adults and alleviate social challenges associated with population aging.

## Brief history of aging and aging-associated diseases

**Fig. 1:**
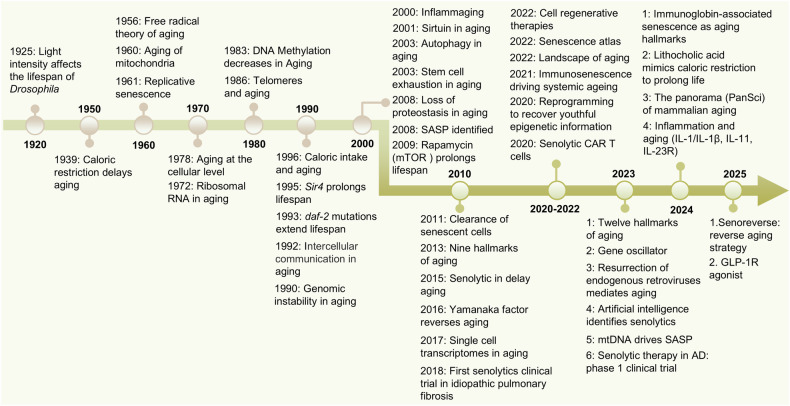
Brief history of aging and its treatment. Over time, research exploration and technological advances have facilitated the development of cognitive and therapeutic approaches for aging, ultimately providing insights into more efficient and precise treatment strategies

The earliest study on aging began in *Drosophila*. In 1925, J H Northrop reported that light can affect the aging rate of *Drosophila*.^8^ In 1939, caloric restriction was subsequently reported to have extended the lifespan of rats and mice,^9^ which was the first evidence that the aging process was regulated (Fig. 1). In 1961, Leonard Hayflick demonstrated that human fetal cells have 50 to 60 generations of limited replication potential, which was subsequently termed the “Hayflick limit” or replicative senescence.^10^ In 1993, Cynthia Kenyon reported that *daf-2* mutations can significantly prolong the lifespan of *Caenorhabditis elegans* (*C. elegans*),^11^ demonstrating for the first time that a single gene can regulate lifespan. Further studies in *Drosophila*, nematodes, and mice have confirmed the conserved role of inhibiting the insulin/insulin-like growth factor-1 signaling (IIS) pathway in prolonging lifespan.^12^ In 1995, a genetic screen confirmed that sir4 regulates the longevity of *S. cerevisiae*.^13^ Seven sirtuin (SIRT1-SIRT7) genes were subsequently discovered, all of which are significantly associated with lifespan and age-related diseases.^14^ The nineties witnessed the discovery of the mammalian target of rapamycin (mTOR) as the molecular target of rapamycin, a conserved kinase integrating nutrient sensing with growth control. Subsequent studies established mTOR inhibition as a key geroprotective mechanism extending lifespan across species, including yeast, worms, flies, and mice.^15^ With the emergence of gene therapy, in 2006, Yamanaka et al. created induced pluripotent stem cells (iPSCs) by introducing four transcription factors (OSKM: Oct4, Sox2, Klf4, and cMyc) into mouse fibroblasts.^16^ In 2016, partial reprogramming of OSKM in vivo was confirmed to prolong the lifespan of progeroid mice.^17^ Recent studies have revealed that partial reprogramming can reverse the epigenetic characteristics of aging without eliminating cells.^18–20^ In recent years, with the development of single-cell sequencing and multiomics, a variety of aging clocks^21^ and aging panoramas^22,23^ have emerged. Studies have also elucidated at the preclinical level the antiaging mechanisms of various clinically established agents, including metformin,^24^ lithocholic acid,^25,26^, and GLP-1R agonist.^27^

Researchers have also discovered and proposed three major treatment strategies for age-related diseases, which encompass compound and genetic-engineering-based strategies: senolytics, senomorphics and senoreverse (Fig. 1). Senolytics are compounds that can selectively eliminate senescent cells. The concept of senolytics was first proposed in 2015 by James Kirkland’s group at the Mayo Clinic,^28^ who investigated the use of a combination of dasatinib and quercetin (D + Q) to eliminate senescent cells. Other agents, such as ABT263,^29^ fisetin,^30^ and 753b (BCL-xL/BCL-2 PROTAC degrader),^31^ were subsequently developed. Clinical trials have shown their potential in the treatment of idiopathic pulmonary fibrosis (IPF, 2018),^32^ Alzheimer’s disease (AD, 2023, 2024, 2025),^33–35^ diabetic macular edema (DME, 2024),^35^ etc. In 2020, senolytic CAR-T-cell therapy was proposed for the precise elimination of senescent cells, showing strong therapeutic ability for aging-related diseases.^36^ Senomorphics are compounds that can inhibit the senescence-associated secretory phenotype (SASP), such as rapamycin and metformin. The SASP was first proposed and summarized by Judith Campisi’s group in 2008.^37^ The senoreverse is a strategy for reversing aging. In 2002–2024, the researchers at Altos Laboratories proposed a strategy of intervening in senescent cells through partial reprogramming,^18,19^ demonstrating the potential to trace senescent cells back to earlier states. In 2025, Ji’s team from the Henan Academy of Sciences demonstrated for the first time that cellular senescence could be reversed by senoreverse strategy.^38^ Collectively, all these discoveries have contributed not only to our understanding of the biological aging process, but also allowed to develop rationale intervention strategies to target molecular hallmarks of this process and biomarkers to quantify biological aging status, aiming to achieved healthy lifespan extension with precise and multitargeted approaches.

## Key hallmarks and molecular mechanisms of aging

**Fig. 2:**
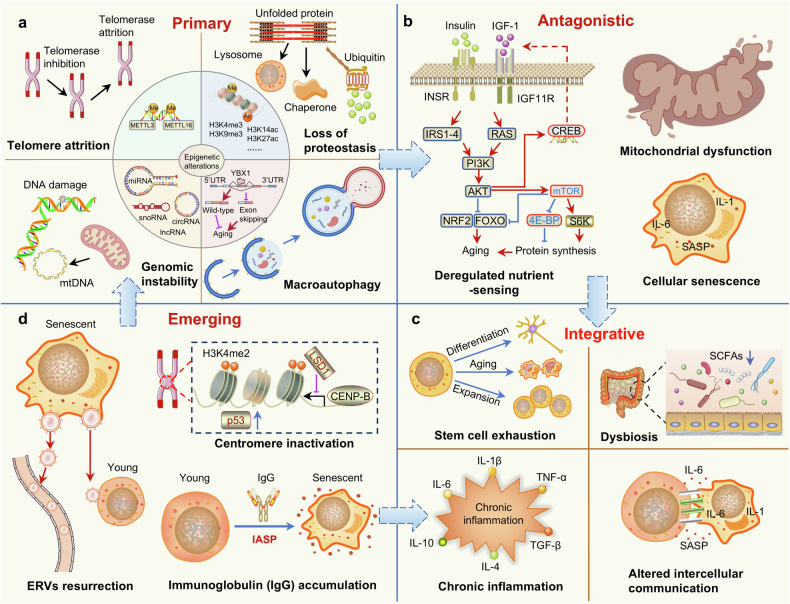
Hallmarks of aging. These hallmarks are subdivided into three categories: **a** five primary features (genomic instability, telomere attrition, epigenetic alterations, loss of proteostasis, and disabled macroautophagy), which are the main and negative causes of cell damage; **b** three antagonistic characteristics (deregulated nutrient sensing, mitochondrial dysfunction, and cellular senescence), which alleviate the damage caused by the primary hallmarks in the early stages but eventually become harmful themselves; **c** and four integrative characteristics (stem cell exhaustion, altered intercellular communication, chronic inflammation, and dysbiosis), which compensate for damage repair caused by primary and antagonistic hallmarks. **d** Emerging aging characteristics include the accumulation of Immunoglobulin G (IgG), reactivation of endogenous retroviruses, and centromere inactivation. SASP, senescence-associated secretory phenotype, IASP immunoglobin-associated senescence, ERVs endogenous retroviruses. Some elements are derived from BioGDP (https://biogdp.com/)

In 2023, researchers integrated and elaborated on 12 characteristics of aging,^39^ including genomic instability, telomere attrition, epigenetic alterations, loss of proteostasis, disabled macroautophagy, deregulated nutrient-sensing, mitochondrial dysfunction, cellular senescence, stem cell exhaustion, altered intercellular communication, chronic inflammation, and dysbiosis.^39^ More recently, extracellular matrix changes and psychosocial isolation have been added to the list.^40^ These characteristics of aging are closely related to each other. Integrative aging characteristics emerge when the cumulative impairment caused by primary and antagonistic aging characteristics fail to be repaired, resulting in stem cell exhaustion, chronic inflammation, dysbiosis and altered intercellular communication,^39^ all of which collectively determine the speed of aging (Fig. 2). In recent years, Liu group has made breakthroughs in understanding the characteristics of aging by discovering two novel hallmarks of aging: immunoglobin-associated senescence^22^ and endogenous retroviruses resurrection.^41^ However, aging is heterogeneous and dynamic, exhibiting different characteristics across various tissues in different aging-related diseases.^42^ An in-depth understanding of the characteristics, regulatory mechanisms and dynamic changes associated with aging can provide us with precise antiaging strategies to prevent aging-related diseases.

### Primary hallmarks-primary molecular damage of aging

Primary characteristics are fundamental to the aging process and represent the intrinsic mechanisms that directly cause cellular and molecular damage and drive aging. These include genomic instability, telomere attrition, epigenetic alterations, loss of proteostasis, and disabled macroautophagy (Fig. 2a).

#### Genomic instability

Genomic instability is one of the central mechanisms of aging, which involves DNA sequence changes, accumulation of damage and mutations, and mitochondrial DNA (mtDNA) release. This DNA damage and mutation affect gene expression and function, leading to cell and organ aging. Genomic instability is also closely related to the occurrence and development of aging-related diseases, such as cancer and neurodegenerative diseases.

##### Nuclear DNA damage

**Fig. 3:**
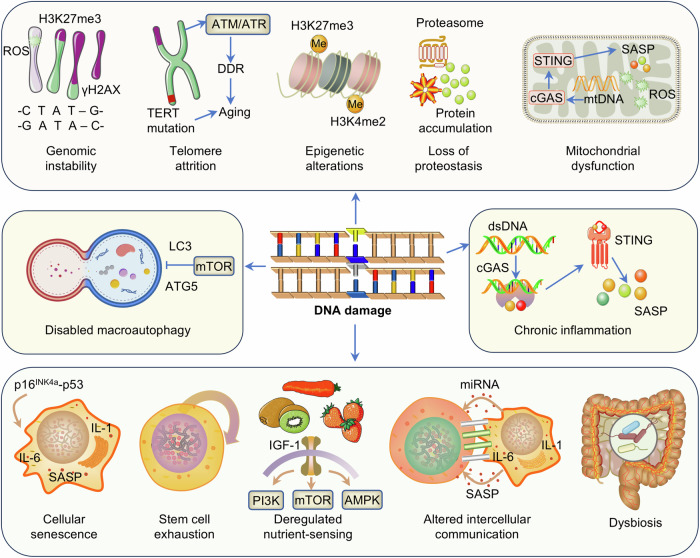
DNA damage forms a multidimensional interaction with aging hallmarks through a complex molecular network. Endogenous reactive oxygen species (ROS) and exogenous toxins continuously attack DNA, leading to mutation accumulation and chromosomal structural abnormalities, accelerating genomic instability. The process of DNA repair is accompanied by dynamic chromatin remodeling, resulting in abnormal methylation of epigenetic marks (such as H3K4me2 and H3K27me3). DNA damage and telomere shortening accelerate aging through a bidirectional feedback mechanism: telomere shortening triggers the DNA damage response, whereas DNA damage under oxidative or replication stress further disrupts telomere stability. DNA damage disrupts mRNA synthesis by blocking transcription, leading to imbalances in protein synthesis and the accumulation of misfolded proteins. The DNA damage response activates proteasome inhibition and autophagy dysregulation, exacerbating amyloid deposition. DNA damage-induced mTOR activation inhibits autophagy initiation, leading to mitochondrial damage and impaired protein aggregate clearance. Senescent cells release exosomes through the SASP, which transmit miRNAs and damage-associated molecular patterns to induce inflammation and epigenetic reprogramming in neighboring cells. Some elements are derived from BioGDP (https://biogdp.com/)

Nuclear DNA damage includes single-strand breaks, double-strand breaks, base oxidation, chromosomal translocations, and aneuploidy. This damage is caused mainly by endogenous factors (such as reactive oxygen species and DNA replication errors) and exogenous factors (ultraviolet radiation, ionizing radiation, and chemical toxins).^39,43^ Studies have confirmed that the efficiency of DNA repair pathways, such as base excision repair, nucleotide excision repair and homologous recombination, significantly decreases with age, resulting in damage that cannot be repaired in time. Unrepaired DNA damage activates inflammatory pathways such as the NF-κB pathway, releases proinflammatory factors (IL-6 and TNF-α), and promotes systemic inflammation and disease progression.^43^ In addition, genetic defects in DNA repair pathways may directly lead to the development of premature aging syndromes, including Werner syndrome,^44^ Bloom syndrome,^45^ Hutchinson-Gilford progeria syndrome^46^, and Cockayne syndrome.^47^ A growing number of studies have confirmed that DNA damage can trigger multiple aging hallmarks and is considered to be the core driver of aging.^43^ DNA damage is not only the initial factor of aging but also the hub connecting various aging characteristics (Fig. 3). It forms a multilevel positive feedback loop by directly destroying genetic information, indirectly regulating epigenetic signaling networks, and triggering autonomous and involuntary cellular effects. DNA damage forms a positive feedback loop that drives aging by interfering with chromatin structure, altering epigenetic marks and causing protein imbalance. In summary, DNA damage serves as the core driving factor, forming a network with other hallmarks to jointly promote the aging process. Collaborative blocking of this network process can provide novel directions for extending a healthy lifespan.

##### Mitochondrial DNA

mtDNA is a circular double-stranded DNA molecule that is independent of the nuclear genome. Its high copy number and lack of histone protection make it more vulnerable to endogenous attack by reactive oxygen species (ROS).^48^ The repair of mtDNA is much less efficient than that of nuclear DNA, resulting in the accumulation of damage.^49^ mtDNA point mutations and deletions increase exponentially with age, disrupt the assembly of the oxidative phosphorylation complex, reduce adenosine triphosphate (ATP) and increase electron leakage, and form a vicious cycle of “ROS-damage-ROS”.^50,51^ This energy deficiency triggers the collapse of the mitochondrial membrane potential (*ΔΨm*) and the release of proapoptotic factors, which activate the caspase cascade and induce apoptosis or senescence.^52^ In clinical studies, mtDNA mutations in aging tissues (such as skeletal muscle and neurons) are significantly increased.^53^ Notably, there is bidirectional communication between mtDNA and the nuclear genome: nuclear-encoded mitochondrial proteins regulate mtDNA replication and packaging, whereas mitochondrial metabolites affect nuclear gene expression through epigenetic modification, forming a “mitochondrial-nuclear feedback loop”.^54^ In summary, mtDNA damage is not only the driving factor of aging but also its hallmark consequence, which promotes a decline in body function through multidimensional mechanisms such as oxidative stress, inflammatory signaling, and epigenetic remodeling. Future researches are needed to integrate multiple omics approaches to analyze the spatiotemporal dynamics of mtDNA mutations and develop precise delivery systems for targeted interventions.

#### Telomere attrition

An increasing number of studies have indicated that telomerase activity and telomere length are strongly linked to aging. Telomere dysfunction can lead to the aging of multiple tissues and organs, as well as the occurrence of age-related disorders,^55^ including AD, Parkinson’s disease (PD), IPF, and age-related macular degeneration. There is a significant negative correlation between telomere length and the aging rate in different species, with some long-lived species having longer telomere lengths and short-lived species having shorter telomere lengths. Normal aging in mammals is accompanied by telomere attrition and the accumulation of shorter telomeres.^56^ The lifespan of organisms can be influenced by interference with telomere length.

Telomere-related DNA damage response (DDR) foci, also known as telomere-induced DNA damage foci, are formed when the DDR is activated at telomeres.^43^ This leads to a lower efficiency of damage repair at telomeres, which accumulates and accelerates a senescence-like phenotype.^55^ Telomerase activity is regulated by the telomerase reverse transcriptase (TERT) gene. Therapeutically, telomere shortening can be inhibited by activating endogenous TERT expression or inducing telomerase activity. The agents that activate telomerase are considered potent antiaging agents for treating age-related diseases. The TERT activator TAC can upregulate TERT transcription and promote telomere synthesis, thereby delaying aging.^57^ When telomerase is inactive in cells, antigen-presenting cells transfer telomeres to recipient T cells through extracellular vesicles (EVs), which can extend the telomere length of T cells more than 30-fold, thereby protecting recipient T cells from replicative aging and prolonging the lifespan of the immune system.^58^ SIRT6 is known to have chromatin regulatory activity in telomeres, and deletion of SIRT6 causes genomic instability and premature cell senescence in primary human fibroblasts due to telomere dysfunction. SIRT6 also maintains repressive heterochromatin in subtelomeric regions, thereby silencing the transcription of telomere genes.^59^ SIRT6 is critical for maintaining the sustained self-renewal capacity of hematopoietic stem cells (HSCs) and human mesenchymal stem cells (hMSCs).^60^ Importantly, lifestyle regimen including diets rich in vegetables, fruits, legumes, nuts, and other foods rich in fiber and antioxidants is also linked to increased telomere length. While interventions such as telomerase activation have not yet fully overcome safety-related bottlenecks, an in-depth understanding of the telomere-aging axis has provided new directions for precision antiaging strategies and disease prevention. Future research should focus on screening agents that can specifically prolong telomeres and activate telomerase and on means to support telomere maintenance through judicious selection of foods.

#### Epigenetic alterations

**Fig. 4:**
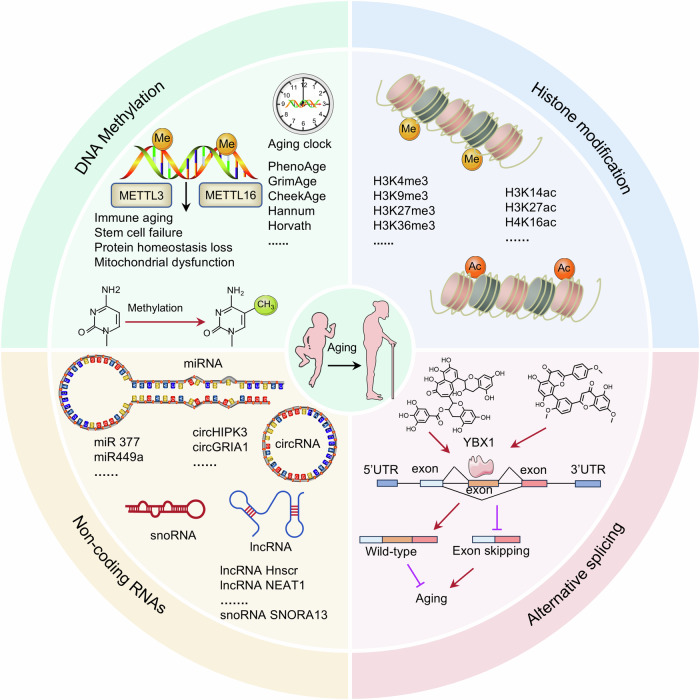
Epigenetics is involved in the regulation of aging. Together, DNA methylation, histone modification, noncoding RNA and alternative splicing constitute an epigenetic network that regulates aging. Some elements are derived from BioGDP (https://biogdp.com/)

Epigenetics refers to the modification of genes without altering the DNA sequence, including DNA and RNA methylation, histone modification, and noncoding RNA.^61^ The loss of epigenetic information has proven to be one of the reasons for the aging of mammals.^62^ Increasing evidence suggests that regulating DNA methylation, histone modifications, RNA splicing and noncoding RNAs in epigenetics (Fig. 4) can delay aging phenotypes.^39,61^ Strategies that focus on the manipulation of epigenetic processes have reduced aging or extended lifespan in animal models.

##### DNA methylation

DNA methylation is the process of obtaining methyl groups through the covalent binding of specific DNA sequences under the action of DNA methyltransferase. This is a core epigenetic process that regulates development, tumorigenesis, and aging. Several DNA methylation-based epigenetic clocks have been established in the genome, such as Horvath, Hannum, PhenoAge, CheekAge, GrimAge, iCASDNAmAge, CultureAGE, and the scAge clock.^61,63^ These clocks have the ability to predict chronological age and offer valuable insights into the aging of organisms. Hypermethylation or hypomethylation of DNA in specific regions leads to abnormal repression or activation of genes, thereby promoting the aging process.^61^ DNA methylation is significantly associated with the maximum lifespan in mammals. Mammals with longer lifespans exhibit tidier DNA methylation patterns, whereas species with shorter lifespans exhibit softer and flatter methylation patterns.^64^ The mechanism of DNA methylation-induced senescence is strongly linked to protein homeostasis loss, mitochondrial dysfunction, stem cell failure, and immune aging. Owing to the genetic diversity of different species, identifying the dynamics of DNA methylation during aging remains challenging. Further insights are needed to understand how age-related DNA methylation regulates gene expression, biological function and lifespan. The next priority is to demonstrate that at least some age-related DNA methylation changes directly cause alterations in the cellular/tissue transcriptome, thereby leading to aging.

##### Histone modification

Histone methylation mainly occurs at the N-terminal lysine or arginine residues of histones H3 and H4, which are regulated via histone methyltransferases, including histone lysine methyltransferase and protein arginine methyltransferase. H3K4me3, H3K9me3, H3K27me3, H3K36me3, H3K4me3, and H4K20me3 participate in gene silencing and heterochromatin formation.^61^ In nematodes, depletion of H3K4me3 activates stress-response genes, which increases stress resistance and prolongs lifespan.^65^ S-adenosylmethionine (SAM) is the only methyl donor that modifies histones, and a reduction in SAM causes a significant decrease in overall H3K4me3 and heterochromatin loss.^66^ Metformin-induced SAM restriction significantly reduces H3K4me3 levels and prolongs lifespan in *C. elegans*.^67^ Interestingly, the functional interaction between DNA methylation and histone modification is crucial for sustaining heterochromatin homeostasis and stem cell genomic stability.^68^ In conclusion, the regulation of histone methylase and demethylase activities may provide beneficial effects for delaying aging.

Histone acetylation undergoes significant changes in aging-related processes. Sirtuins are a family of nicotinamide adenine dinucleotide (NAD^+^)-dependent deacetylases composed of seven proteins, SIRT1-SIRT7, which are highly conserved among species. Sirtuins are known as longevity proteins because of their noteworthy roles in stress resistance, metabolism, apoptosis and aging. SIRT1 is considered to be a longevity protein that participates in the modulation of delayed premature aging. La ribonucleoprotein domain family member 7 (LARP7) is an allosteric agonist of SIRT1 that combines with the N-terminus of SIRT1 to regulate the activity of SIRT1 deacetylase, thereby modulating aging and atherosclerosis, and its activating effect is superior to that of AROS.^69^ DNA damage mediates ataxia telangiectasia mutated activity and reduces SIRT1 deacetylase activity by disrupting the LARP7-SIRT1 interaction, thereby accelerating cell senescence.^69^ In addition to the sirtuin family of deacetylases, other deacetylases and acetyltransferases are also strongly associated with aging, including HDAC1,^70^ CBP-1,^71^ KAT6A/B,^72^ and SAS2.^73^ In conclusion, the regulation of histone acetylation can provide a new direction for developing aging-delaying approaches.

##### Alternative splicing and noncoding RNAs

Alternative splicing (AS) is the process of generating alternative mRNA splicing isoforms from an mRNA precursor via various splicing modes. AS plays a significant role in increasing proteome diversity and controlling gene expression. AS appears in transcripts generated by approximately 95% of human genes, including genes related to aging, apoptosis, and DNA repair.^74^ The splicing of pre-mRNAs is fundamental for linking gene expression to the proteome, and splicing dysregulation has been connected with age-related chronic diseases. Pre-mRNA splicing homeostasis is a hallmark of life expectancy in *C. elegans*, and splicing factor 1 extends lifespan through dietary restriction and modulation of the AMPK, RAGA-1 and RSKS-1/S6 kinases in the TORC1 pathway.^75^ Furthermore, the lncRNAs MIR99AHG,^76^ CTC-490G23.2,^77^ DCRT,^78^ Pnky,^79^ and miR-124^80^ can directly target PTBP1 to regulate the AS network. We summarize the most promising noncoding RNAs that have been shown to regulate aging in recent years (Table 1) to provide a basis for screening agents that target lncRNAs to delay aging.

**Table 1:** Important aging-related noncoding RNAs identified in recent years

Targets	Types of aging	Effects on aging	Treatment strategies	Ref.
miR-31	Skin aging	Negative regulation	Knockout/MAPK/ERK inhibitors	^478^
miR-377	Skin aging	Negative regulation	Inhibitors	^479^
miR-29c-3p	Systemic aging	Negative regulation	—	^480^
miR-29	Skeletal senescence	Negative regulation	Knockdown	^481^
miR-7	Cell senescence	Negative regulation	Knockdown/Knockout	^482^
miR-449a	Cell senescence	Positive regulation	miR-449a mimetics	^483^
miR-708	Cell senescence	Positive regulation	Overexpression	^148^
LncRNA CYTOR	Skeletal muscle aging	Positive regulation	Adeno-associated virus serotype 9 delivery of CRISPRa	^484^
LncRNA METTL3-m^6^A	Skeletal muscle aging	Positive regulation	Overexpression	^485^
LncRNA NEAT1	Skeletal aging	Negative regulation	Extracellular vesicle delivery	^486^
LncRNA SNHG12	vascular aging	Positive regulation	Intravenous injection	^487^
LncRNA Altre	Liver aging	Positive regulation	—	^488^
LncRNA LINE-1	Progeroid syndromes Cell senescence	Negative regulation	Antisense oligonucleotide	^212,213^
LncRNA H19	Endothelial cell senescence	Positive regulation	—	^489^
LncRNA KCNQ1OT1	Cell senescence	Positive regulation	—	^490^
LncRNA MAGI2-AS3	Cell senescence	Negative regulation	Knockdown	^491^
hSATII RNA	Cell senescence	Negative regulation	Knockdown	^492^
LncRNA TERRA	Cell senescence	Positive regulation	—	^493^
LncRNA FRAIL1	Skeletal muscle aging	Negative regulation	Knockdown	^494^
circSfl	Aging	Positive regulation	Overexpression	^495^
circFoxo3	Cardiac senescence	Negative regulation	Knockout	^496^
circGRIA1	Brain aging	Negative regulation	Knockdown	^497^
circHIPK3	Cardiac senescence	Positive regulation	UMSC-Exos delivered circHIPK3	^498^
circPVT1	Endothelial cell senescence	Positive regulation	Overexpression	^499^
circKIF18A	IDD	Positive regulation	Injection of adenoviral circKIF18A	^500^
circATXN1	IDD	Negative regulation	siRNA nucleic acid framework delivery system	^501^
circRREB1	Chondrocyte senescence	Negative regulation	Injection of adenovirus-CircRreb1	^502^
snoRNA SNORA13	Cell senescence	Negative regulation	CRISPR	^503^

#### Loss of proteostasis

Proteostasis refers to the dynamic balance of a complex network of proteins in cells, including protein synthesis, structural and functional maturation, protein quality control, folding modification, localization and transport, functional recovery and degradation.^81^ This balance is achieved through the synergistic effects of the molecular chaperone system, ubiquitin-proteasome system (UPS) and autophagy-lysosome system. The imbalance of proteostasis caused by aging is characterized by decreased protein synthesis and folding efficiency, dysfunction of the protein degradation system, and abnormal posttranslational modifications.^81,82^ Together, these changes aggravate the accumulation of misfolded or abnormal proteins, triggering cellular dysfunction and even leading to age-related pathological states.

##### Protein synthesis and folding

The ER is the main organelle for protein and lipid synthesis within cells, where almost all lipids and various important proteins are synthesized.^83^ Protein disulfide isomerase (PDI), a key molecule involved in protein oxidative folding, is upregulated in multiple model organisms.^84^ During the oxidative folding process of endoplasmic reticulum (ER) proteins, the formation of disulfide bonds produces the byproduct H_2_O_2_, which is released into the nucleus to modulate the level of the aging-related gene SERPINE1 and promote cell senescence.^85^ PDI knockout can decrease the rate of protein oxidative folding and delay stem cell senescence.^85^ On the other hand, the increase in nitrosylation of Cys166 and Cys131 in the ER thiol oxidase Ero1α leads to a decrease in oxidase activity with aging, stimulating ER reductive stress. ER proteostasis and ER-unfolded protein response (UPR) capacity are reduced under ER reductive stress, thereby promoting senescence.^86^ Hence, delaying aging through the inhibition of the ER protein redox-induced folding process is a novel approach.

##### Dysfunction of the protein degradation system

The protein degradation system, including the UPS and autophagy-lysosome pathway (ALP), is the core mechanism involved in maintaining proteostasis in cells.^87^ During aging, the function of these systems is progressively dysregulated, leading to the accumulation of misfolded or damaged proteins, which in turn triggers cellular dysfunction and aging-related diseases.^88^ The UPS is involved in the degradation of more than 80% of endogenous proteins in cells and plays an important role in regulating cell function.^89^ However, the degradation efficiency is significantly reduced during aging owing to oxidative stress and substrate overload.^88^ Oxidative damage to proteasomes in neurons is closely related to the aggregation of Aβ and tau proteins in the brains of patients with AD.^90^ In the UPS, the E3 ligase is considered an important component of the ubiquitin binding system and plays a role in substrate-specific recognition. Therefore, proteasome activation can intervene in aging and age-related diseases. For the maintenance of protein homeostasis by the ALP, we elaborate on autophagy and aging in the next section.

The UPS and ALP are not independent pathways; they can synergistically regulate protein homeostasis.^89,91^ Proteases cooperate with the autophagy-lysosome system and the UPS to regulate protein homeostasis in aging and age-related neurodegeneration.^92^ Proteasome activity is reduced in the brains of patients with AD^93^ or PD,^94^ whereas accumulation of the autophagy marker p62 is positively correlated with cognitive decline.^95^ Future studies need to combine single-cell transcriptomics and ubiquitin-modified omics to analyze the dynamic regulatory network of the proteasome, UPS and ALP during aging to ensure the correct synthesis, folding, and degradation of proteins, effectively ameliorating age-related pathologies.

#### Autophagy disruption

**Fig. 5:**
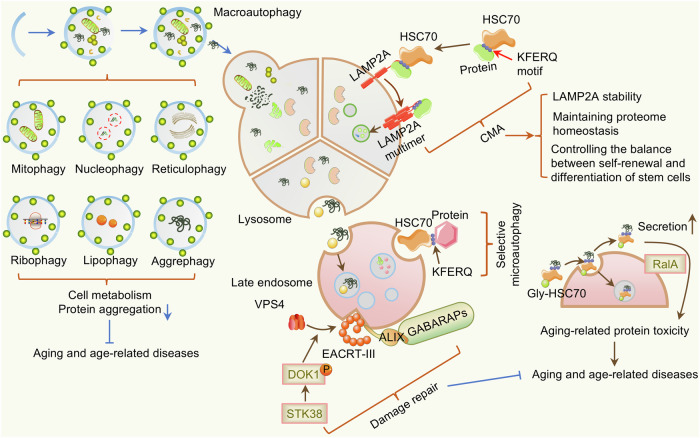
Autophagy regulates aging. Macroautophagy, chaperone-mediated autophagy, and microautophagy can delay aging in a variety of ways, including maintaining protein homeostasis, removing age-related toxic proteins, and repairing damaged cell membranes. Other types of autophagy, such as mitophagy, nucleophagy, lipophagy, ribophagy, aggrephagy, and reticulophagy, can also delay aging by regulating cell metabolism and protein aggregation. Some elements are derived from BioGDP (https://biogdp.com/)

Autophagy is an evolutionarily conserved catabolic homeostatic program for intracellular material turnover in eukaryotes. Autophagy is separated into three types according to the way in which intracellular substrates are transported to lysosomes: macroautophagy, microautophagy, and chaperone-mediated autophagy (CMA)^96^ (Fig. 5). Autophagy directly or indirectly participates in the regulation of aging in various eukaryotes. The function of autophagy gradually weakens with age, and the failure of autophagy gene function further leads to a shortened lifespan of the organism, forming a vicious cycle. Enhancing autophagy (physiological inducers or pharmacological inducers) helps extend the lifespan and healthy lifespan of mammals.^97^

Accumulating evidence indicates that autophagic activity decreases with age in multiple organisms and that the material transport of lysosomes decreases, indicating that damaged autophagy is a canonical characteristic of aging in organisms. The breakdown of protein homeostasis (misfolding, mislocalization, and aggregation of proteins) is a core characteristic of aging. Although age-related protein homeostasis loss has been recorded in many tissues, age-dependent protein aggregation is closely associated with neurodegenerative diseases. By removing harmful misfolded and aggregated proteins, autophagy supports a healthy aging process. In 2003, the autophagy gene *bec-1* (mammalian beclin1) was revealed to be indispensable for longevity extension in nematodes with dysfunction in the IIS pathway.^98^ Subsequently, functional analysis of multiple autophagy-related genes revealed the crucial role of autophagy in extending lifespan. Activating autophagy and increasing autophagic flux *via* nutritional, pharmacologic and genetic manipulations that contribute to the extension of longevity.^99^ Even so, authoritative evidence that enhancing basal autophagy can prolong the healthspan and lifespan of mammals was not discovered until 2018. Specifically, disruption of beclin1-BCL2 binding is associated with increased levels of basal autophagic flux. The decrease in beclin1-BCL2 binding is linked to longevity and improved aging-related phenotypes.^100^ Autophagy-related gene (ATG) family members (ATG5, ATG7, ATG8, etc.) are the core components of autophagy and are essential for the initiation and execution of autophagy. Knockout of ATG5 or ATG7 can shorten the lifespan of experimental animals, whereas overexpression of ATG5 can prolong the lifespan.^101^ As a selective autophagy receptor, p62/SQSTM1, when upregulated in nematodes and *Drosophila*, can promote protein homeostasis and extend lifespan.^101^ Transcription factor EB (TFEB, HLH-30 in *C. elegans*) is a transcriptional regulator of autophagy and lysosome biogenesis, and TFEB nuclear translocation is reduced during aging, resulting in a decrease in lysosomal number and activity. TFEB activators, such as rapamycin, reverse the age-related decline in autophagy-lysosomal function, and overexpression of HLH-30 prolongs the lifespan of *C. elegans*.^102^ Autophagy can delay aging in a variety of organs, including cardiac aging,^103^ vascular aging,^104^ brain aging,^105^ skin aging,^106^ skeletal muscle aging,^107^ liver aging,^108^ eye aging,^109^ and systemic aging.^110^ Furthermore, other types of autophagy, such as nucleophagy and lipophagy (Fig. 5), have also been found to regulate aging. Nucleolar size is linked to longevity and life-prolonging interventions, and small nucleoli represent a biological signature of longevity.^111^ Abnormal nuclear anchor protein 1 (ANC-1)/nesprin-2 is eliminated and restrict nucleolus size through autophagy. Furthermore, nucleophagy contributes to stress resistance, germline immortality and longevity.^112^

CMA is a type of autophagy that selectively degrades substrates carrying KFERQ motifs through lysosomes. CMA activity decreases with age in the majority of tissues and organs, which is due to the decreased stability of lysosomal-associated membrane protein 2A (LAMP2A) on the lysosomal membrane of aging organisms.^113^ There are multiple pathways by which CMA dysfunction induces senescence, including the generation of DNA damage, cell cycle arrest (expression of *p12/Cdkn1a*), and lipid accumulation.^114^ The first direct evidence of aging amelioration effects by activating CMA was demonstrated in a double transgenic mouse model. CMA enables improvement of intracellular protein disorders and maintenance of hepatic function in aging.^115^ CA, a small-molecule agonist of CMA, increases LAMP2A receptor levels on lysosomes to improve AD.^116^ Additionally, caloric restriction can mediate constitutive activation of CMA to improve health and promote healthy aging.^117^ Considering the core role of CMA in maintaining cellular homeostasis and delaying aging, intervention strategies targeting CMA may provide new strategies for the treatment of age-related diseases.

Compared with those of macroautophagy and CMA, the molecular mechanism and physiological implications of microautophagy are still unclear, but the basic characteristics of microautophagy in aging have been revealed. Microautophagy activity decreases with age and is related to an increase in protein secretion. Serine-threonine kinase 38 (STK38/NDR1) is specifically recruited to damaged lysosomes and, together with gamma-aminobutyric acid receptor-associated protein (GABARAPs), repaired damaged membranes through microautophagy.^118^ Maintenance of lysosomal integrity through the modulation of microlysophagy by STK38 and GABARAPs presumably contributes to preventing aging.

Given the core role of autophagy in aging and the dependency of many geriatric protective agents on autophagy, identifying direct regulators of autophagy that contribute to delaying aging and prolonging a healthy lifespan is critical. At present, research on agents that target autophagy for antiaging mainly focuses on macroautophagy, whereas few studies have focused on natural products that activate chaperone-mediated autophagy and microautophagy, which need to be further explored.

### Antagonistic hallmark-compensatory responses

The abovementioned primary initial injury triggers adaptive responses in cells. Initially, such responses can alleviate injury, but long-term overactivation can turn them harmful, thereby leading to compensatory reactions, namely, antagonistic characteristics of aging. These include dysregulation of nutrient sensing, mitochondrial dysfunction, and cellular senescence (Fig. 2b).

#### Deregulated nutrient-sensing

The nutrient-sensing signaling pathway is highly conserved in different species. It is the ability of cells to perceive and respond to energy metabolism substrates. Extensive evidence has demonstrated that the inhibition of nutrient-sensing pathways contributes to delaying the aging process in organisms. We focus on three key nutrient-sensing pathways, including the mTOR, IGF-1, and adenosine monophosphate (AMP)-activated protein kinase (AMPK) pathways.

##### Insulin/IGF-1 signaling pathway

**Fig. 6:**
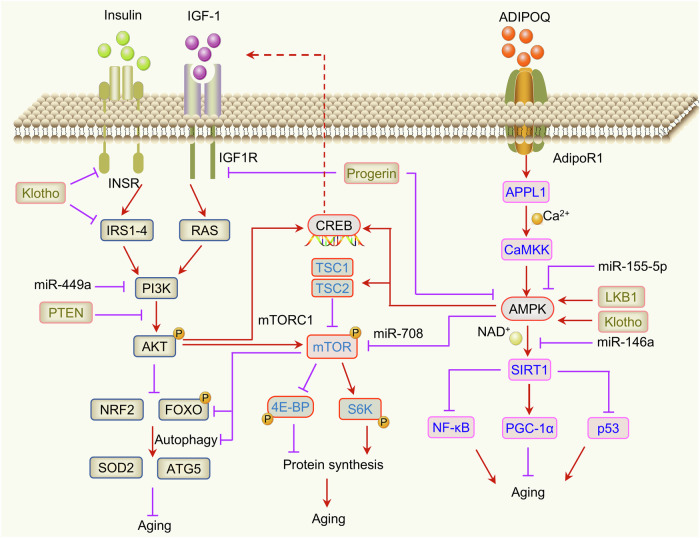
Nutrient-sensing signaling networks regulate aging. The three regulatory networks involved in nutrient sensing are interrelated in the regulation of aging. IGF-1 promotes aging by activating the PI3K-AKT pathway and inhibiting NRF2/FoxO-induced autophagy. Moreover, it can also cross-link to the mTOR signaling pathway through AKT. mTOR induces aging by inhibiting autophagy, promoting protein synthesis, and destroying protein homeostasis. mTOR forms a regulatory network with AMPK through TSC1/TSC2 and miR-708 to jointly regulate aging. AMPK inhibits aging by activating SIRT1 and downstream signaling pathways. LKB1, Klotho, progerin and miRNA participate in the regulation of aging through AMPK

The IIS pathway (Fig. 6) is the first identified and widely validated signaling pathway that regulates aging.^119^ Reducing insulin signaling activity significantly prolongs the lifespan of *C. elegans*,^11,119^ *Drosophila*,^120^ and mice.^121,122^ Moreover, it can also alleviate the pathology of age-related diseases, such as AD and PD.^123,124^ The analysis of aging-related genes in mammalian evolution reveals that the natural longevity of mammals is strongly linked to the IIS pathway and immune response.^125^ Mutation or deficiency of the *daf-2* (IGF-1) gene can double the longevity of *C. elegans*.^11^ DAF-2/DAF-16 (FOXO) signaling in the gut contributes greatly to longevity regulation, with minor effects on the hypodermis, muscle, and reproductive system.^124,126^ The reduction in IIS activity in the intestine can almost double the lifespan of nematodes without affecting development and reproduction.^124^ In humans, IGF-1 levels and the mortality hazard ratio have a U-shaped association according to the meta-analysis. IGF-1 levels between 120 and 160 ng/mL are associated with the lowest mortality risk, and both high and low levels increase the risk of mortality.^127^ Interestingly, increasing IGF-1R in young mice results in superior cardiac repair function. While activation of IGF1R during aging results in poorer cardiac function and shortened lifespan.^128^ IGF1R exacerbates cardiac dysfunction through inhibiting autophagy and mitochondrial oxidative capacity in elderly individuals. Inhibition of IGF1R can effectively promote heart health and prolong lifespan.^129^ These findings provide further evidence that the association between the IGF1R pathway and heart health is not linear but rather bidirectional. The precise regulatory mechanism by which IGF-1 regulates lifespan and healthy lifespan requires further research.

##### mTOR signaling pathway

mTOR is a validated target strongly associated with aging (Fig. 6). The link between mTOR signaling and lifespan is highly conserved. Inhibition of the mTOR pathway prolongs lifespan in all species studied to date.^130^ mTORC1 activator content is decreased in long-lived animals, and the downregulation of mTORC1 contributes to mammalian evolution.^131^ As we age, protein synthesis becomes imbalanced and is regulated by the mTOR pathway.^132^ mTOR activation leads to excessive protein synthesis, which results in impaired protein homeostasis and accelerated aging.^133^ Importantly, S6K1 depletion, which is a component of mTOR signaling, also leads to a prolonged lifespan in mice.^134,135^ Phase 2b and phase III clinical studies have revealed that mTOR inhibitors (RTB101) can improve the immune capacity of the elderly.^136^ Therefore, the immune effects of mTOR inhibitors in prolonging lifespan and healthspan deserve further investigation.

Suppression of the TORC1 gene in nematodes activates SKN-1 and DAF-16, thereby increasing stress resistance and lifespan.^137^ Inhibition of mTORC1 signaling in the liver prevents aging-induced ketogenesis defects and inhibits mouse liver aging.^138^ Rapamycin, an mTOR inhibitor, significantly prolongs the lifespan of mice.^139^ However, long-term administration of rapamycin has significant side effects (including skin and metabolic disorders) and inhibits mTORC2.^140^ Inhibition of mTORC2 has been proven to shorten the lifespan in *C. elegans*^141^ and in liver-specific mTORC2 knockout mice.^142^ Hence, mTORC1-specific inhibitors or intermittent treatments that avoid disrupting mTORC2 or only reduce rather than eliminate mTORC1 pathway activity may constitute safer strategies for treating age-related diseases.

##### AMPK signaling pathway

AMPK is a key mediator of intracellular energy metabolism and an important regulator of nutrient sensing that regulates the biochemical effects of nutrient substances. The responsiveness of AMPK activation decreases during the aging process. Increasing AMPK activity contributes to prolonging longevity in multiple model organisms, including *Drosophila*, nematodes and mice.^143^ Selective activation of AMPK_γ1_ can maintain metabolic balance and promote healthy aging.^144^ PARP1 activity is upregulated in the aging skeletal muscles of elderly individuals, mice, and *Drosophila*.^145,146^ Knocking down PARP1 can extend the longevity of *Drosophila* by regulating PARylation and AMPKα activity.^147^ The interaction between PARP1 and AMPKα inhibits AMPKα activity and mitochondrial turnover.^147^ These findings further reveal the mechanism by which AMPKα regulates mitochondrial homeostasis during aging. Interestingly, AMPK suppressed cell senescence and mouse aging by upregulating the expression of miR-708, which directly targets *Dab2* and inhibits mTORC1 activity.^148^ Therefore, miR-708 is a key regulatory factor connecting the AMPK and mTORC1 signaling networks in aging regulation. AMPK regulates aging through integrating multiple key cell signals, including AMPK-mTOR, AMPK-Nrf2, AMPK-SIRT1 and other regulatory networks (Fig. 6). Although numerous studies have confirmed the pro-longevity effects of AMPK activators, their clinical translational applications are still limited and require further exploration.

The effects of IGF-1, mTOR and AMPK signaling on human nutrition are extremely complex. Although AMPK can prolong lifespan, the biosynthetic pathways required for hormones, cholesterol and other important molecules are impaired when AMPK is chronically activated. In addition, long-term activation of IGF-1 and mTOR can inhibit the AMPK pathway. Therefore, it is necessary but challenging to strictly maintain the dynamic balance among IIS, mTOR, and AMPK activity.

#### Mitochondrial dysfunction

**Fig. 7:**
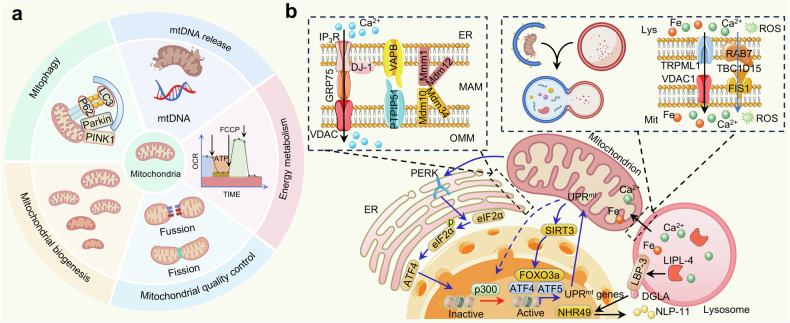
Targeting mitochondria to delay aging. **a** Modulation of mitophagy, mitochondrial biosynthesis, mitochondrial quality control, mitochondrial energy metabolism and mitochondrial DNA release can delay aging. **b** Mitochondria, lysosomes, the endoplasmic reticulum, and internuclear molecular signaling regulate cell senescence. Some elements are derived from BioGDP (https://biogdp.com/)

As a dynamic biophysical system, mitochondria play a major role in regulating energy metabolism and dominating cell death or survival. Research has demonstrated that mitochondrial dysfunction, mitochondrial biogenesis, mitochondrial quality control (MQC), and mitophagy (Fig. 7a) are related to aging.^149^ Mitochondrial dysfunction accelerates T-cell senescence and induces senile changes in metabolic, cognitive and cardiovascular features of aging in mice.^150^ Mitophagy contributes to maintaining mitochondrial homeostasis by selectively removing impaired and aged mitochondria. This process reduces excessive accumulation of ROS, thereby interrupting the vicious cycle of cellular senescence.^149^

MQC ensures cellular homeostasis by coordinating various processes, including protein homeostasis, biogenesis, dynamics, and mitophagy. Preclinical evidence suggests that modulation of MQC can be used to treat cardiac aging, cardiovascular diseases and skin aging.^151^ However, either excessive activation or inhibition of MQC can cause the accumulation of oxidized proteins, expedite abnormal energy metabolism, and induce mitochondrial dysfunction to stimulate aging. Mitochondrial UPR is an important part of MQC, which can effectively remove unfolded or misfolded proteins under stress to maintain the stability and health of mitochondria. Dysregulation of mitochondrial protein folding stress is a precipitating factor for HSC and NSC senescence. Reducing mitochondrial protein-folding stress can improve neurogenesis, the cognitive function of NSCs and the regenerative capacity of HSCs.^152,153^ Minority mitochondrial outer membrane permeabilization (miMOMP) normally occurs in senescent cells, enhances the secretion of mtDNA into the cytoplasm and stimulates the cyclic GMP-AMP synthase-stimulator of interferon genes (cGAS-STING) pathway to promote SASP. Inhibition of miMOMP can reduce the expression of inflammatory factors and ameliorate metabolic homeostasis in aging mice.^154^ In conclusion, targeted regulation of mitochondria can delay aging.

The mitochondria-ER interaction is the central hub of cellular metabolism and regulates the interchange of lipids and metabolites between organelles. The mitochondria-associated membrane or ER-mitochondria contact sites (ERMCSs), which are regions of tight linkage between the ER and mitochondria, are crucial for both cellular physiology and disease (Fig. 7b).^155^ ERMCS are capable of remodeling to satisfy the metabolic requirements of cells in response to alterations in intracellular homeostasis. Disruption of the regional homeostasis of ERMCS affects their remodeling ability, leading to impaired intercellular communication and even the occurrence of ALS.^156^ Inflammation and mitochondrial Ca^2+^ may play significant roles in aging. Research has indicated that the mitochondrial Ca^2+^ uptake capacity of macrophages gradually decreases with age.^157^ ER-mitochondrion calcium transport contributes to regulating mitochondrial Ca^2+^ homeostasis.^158^ Inositol 1,4,5-trisphosphate receptors mediate ER Ca^2+^ release and subsequent mitochondrial Ca^2+^ accumulation, leading to cell senescence.^158–160^ In AD, hyperoside reduces Aβ oligomers by regulating the ER-mitochondrial Ca^2+^ signaling cascade, which can improve learning, spatial memory and motor ability in mice.^161^ At present, there are few studies on such drugs, and the discovery of agents that target interorganelle contacts to delay aging still has much room for exploration.

Cell senescence is largely influenced by the interplay between mitochondria and lysosomes, not merely mitophagy (Fig. 7b). Lysosomal signaling triggers the modulation of mitochondrial activity, which improves metabolic homeostasis, redox homeostasis, and longevity. Mitochondrial-lysosome contacts are mainly involved in organelle dynamics and the exchange of ions and lipids among organelles. In aging-related PD, the number of mitochondrial-lysosomal contacts decreases, and the instability of these contact sites leads to disruption of mitochondrial and lysosomal amino acid homeostasis. These discoveries demonstrate that mitochondrial-lysosomal contact may act as a platform for amino acid transfer between organelles.^162^ Thus, directly regulating interorganelle contacts can be a potential therapeutic means to ameliorate age-related nervous system disorders.

#### Cellular senescence

In 1961, Hayflick first reported that when cell division reaches its limit, it stops dividing and enters a state of irreversible proliferation arrest and proposed the famous “Hayflick limit”.^10^ This is the earliest description of replicative senescent cells. In 2024, the SenNet program summarized nine hallmarks of senescent cells: cell cycle arrest, SASP, nuclear changes, changes in cell surface markers, changes in cell morphology, increases in lysosomal content, metabolic adaptation, DNA damage, and upregulation of antiapoptotic pathway.^163^ A variety of anti-cellular senescence strategies have been developed to address these characteristics. Recent studies have shown that cell cycle arrest is not completely irreversible. By targeting the cell cycle inhibitors *Cdkn1a* and *Ccng2*, miR-302b can release cell cycle arrest, restore the proliferation ability of senescent cells, and significantly reverse the aging phenotype of mice.^38^ This provides a new direction and solution for the treatment of age-related diseases. Unfortunately, cellular senescence can spread not only within the same organ through paracrine signals but also from one organ to another through systemic endocrine signals. Systemic senescence triggered by hepatic senescent cells through transforming growth factor beta (TGFβ) signaling is the main cause of multiple organ dysfunction.^164^ Importantly, depletion of p16^Ink4a^-positive hepatic senescent macrophages significantly attenuated hepatocyte injury.^165^ Blocking TGFβ signaling can effectively mitigate the effects of aging transmission, providing a new direction for the treatment of aging-related diseases.

**Fig. 8:**
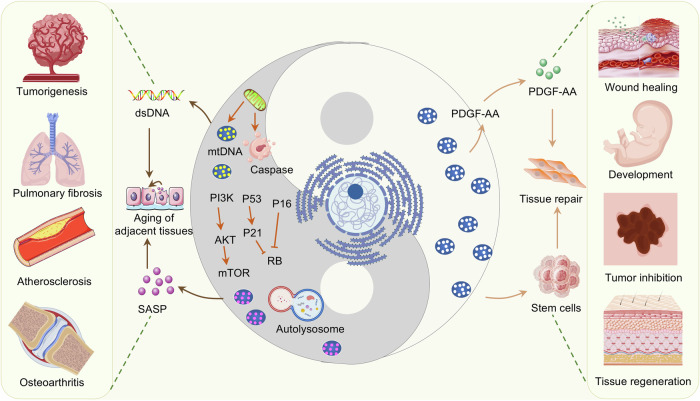
The Yin and Yang of senescent cells. Senescent cells promote the aging of adjacent cells and tissues via the SASP and promote tumorigenesis, pulmonary fibrosis, atherosclerosis, osteoarthritis, etc. On the other hand, senescent cells release platelet-derived growth factor AA (PDGF-AA) and activate tissue stem cells to promote damage repair. Senescent cells can promote wound healing, embryonic development, tissue regeneration, tumor suppression and so on. Some elements are derived from BioGDP (https://biogdp.com/)

Senescent cells can aggravate neurodegenerative diseases,^166^ cardiovascular diseases,^5,167^ atherosclerosis,^168^ osteoarthritis,^169^ etc. Remarkably, not all senescent cells are detrimental (Fig. 8); senescent cells are still involved in many important physiological processes, such as tumor suppression, embryonic development, wound healing and tissue remodeling.^42,170^ Some senescent cells can be inserted into young healthy tissues to defend normal tissues from damage by accelerating adjacent stem cell growth and activating repair.^171^ The senescent cells of fibroblasts and endothelial cells are able to expedite wound healing by stimulating myofibroblast differentiation.^172^ The senescent cells are not replaced after removal but instead disturb the blood-tissue barrier, leading to fibrosis of the liver and perivascular tissues.^173^ Unfortunately, the lack of specificity and severe side effects of existing senolytic therapies limit their potential as therapeutic agents. Although strategies for precise elimination of senescent cells have been reported,^174^ they can only distinguish between senescent cells and healthy cells and cannot distinguish beneficial senescent cells. On the basis of this dilemma, recent research has made breakthroughs. A double recombinase-mediated genetic system (Sn-pTracer, Sn-cTracer and Sn-gTracer)^165^ was generated on the basis of the cell senescence marker p16^Ink4a^. Using this system, it was revealed that depletion of p16^INK4a^-positive macrophages significantly alleviated liver injury, whereas depletion of p16^INK4a^-positive endothelial cells aggravated liver injury.^165^ This system can precisely identify and target cell-type-specific senescent cells, providing insights for the future development of cell type-specific aging therapies. While exciting, the continuous discoveries in the senescence field also highlight that it is necessary to develop superior and more targeted senolytic drugs to eliminate pathological senescent cells while preserving healthy senescent cells. In the future, longevity-extending or antiaging agents should target senescent cells related to diseases rather than regeneration.

### Integrative characteristics

When the compensation mechanisms fail to eliminate the continuously accumulating damage, they lead to the collapse of tissue homeostasis and overall function, thereby exhibiting the characteristics of systemic failure (Fig. 2c).

#### Stem cell exhaustion

The essence of stem cell exhaustion is a reduction in stem cell number, functional degradation and loss of regeneration ability, leading to an imbalance in tissue homeostasis and decreased repair ability. Stem cells can recognize signals released by senescent cells and localize to tissues in need of repair and regeneration, improving the aging state. Hence, the age-dependent decline in the regenerative capacity of stem cells is involved in the aging process. Stem cell exhaustion can lead to the occurrence and development of a variety of age-related diseases. During bone aging, knocking out histone demethylase 4B (KDM4B) in hMSCs induces the formation of age-related heterochromatin foci, thereby impairing the self-renewal of hMSCs and promoting hMSC exhaustion, leading to bone aging and osteoporosis.^175^ Satellite cells in hMSCs are functionally reduced during muscle aging, which in turn impairs cell–cell interactions. Epigenetic changes, telomere shortening and DNA damage, proteotoxicity, mitochondrial dysfunction, and changes in intercellular communication and key signaling pathways (MAPK,^176^ JAK-STAT3,^177^ and Notch^178^) all lead to permanent cell cycle arrest and exhaustion of satellite cell pools.^179^ Activation of autophagy in aged satellite cells can reverse aging and restore stem cell regenerative function.^180^ The escape of hair follicle stem cells (HFSCs) leads to stem cell depletion. The expression of cell adhesion and extracellular matrix genes, which are regulated by Foxc1 and Nfatc1,^180^ is decreased in aged HFSCs. The aged HFSC niche shows extensive alterations in extracellular matrix composition and basement membrane stiffness,^181^ which are central regulators of age-dependent stem cell exhaustion. HSCs are adult stem cells that maintain lifelong hematopoietic function, with self-renewal and multilineage differentiation potential.^182^ The senescence and exhaustion of HSCs are the core reasons for the decline in hematopoietic system function, involving multiple mechanisms such as telomere attrition, oxidative stress, inflammatory disorders and epigenetic disorders, which ultimately lead to anemia, immunodeficiency, and an increased risk of hematological diseases.^183^ The main characteristics of HSC senescence include a decrease in self-renewal ability, an imbalance in differentiation bias, and epigenetic abnormalities.^183^

Stem cell-rich umbilical cord blood infusion has shown efficacy and safety as adjuvant therapy for older adults with acute leukemia in a phase 2 clinical trial (ChiCTR-OPC-15006492).^184^ Despite the widespread use of stem cell therapy, scientists are still struggling to elucidate the molecular mechanism of stem cell depletion. Recent studies have shown that changes in chromosome structure and gene expression may be important causes of stem cell depletion.^66^ In the future, with the development of stem cell research, we are expected to find effective antiaging methods and even reverse some aging-related diseases.

#### Altered intercellular communication

Intercellular communication is a central mechanism for maintaining tissue homeostasis and an important driving force for pathological changes during aging. With increasing age, the intercellular communication network becomes disturbed, leading to the diffusion of inflammatory factors, metabolic disorders and organ dysfunction. Various interactions, such as those involving EVs and soluble factors, play key roles in aging.

##### Exosome-mediated intercellular communication

Exosomes are nanoscale vesicles secreted by cells that carry biological molecules such as proteins and miRNAs and transmit signals to regulate cell functions by fusing with recipient cells or being endocytosed. Noncoding RNAs in exosomal vesicles are promising biomarkers of aging. Senescent bone marrow monocytes/macrophages (BMMs) can spread senescence to multiple tissues by secreting EVs that encapsulate microRNAs, leading to age-related dysfunction.^185^ miR-378a-3p and miR-191-5p play important roles in BMM-EV-mediated systemic aging and age-related dysfunction.^185^ Interestingly, intravenous injection of EVs from young mice stimulated PGC-1α expression through their miRNA cargo, thereby improving mitochondrial function and prolonging the lifespan of aged mice.^186^ Surprisingly, human embryonic stem cell-derived exosomes enriched with miR-302b could rejuvenate aging mice by reversing the proliferation arrest of senescent cells.^38^ In addition, miR-21-5p/miR-217 carried by senescent EVs transmits pro-aging signals, affects DNA methylation and cell replication, and results in senescent phenotypes in recipient cells.^187^ Therefore, EV miRNAs may play a dual role in the aging process: on the one hand, they stimulate cells and tissues to become more dynamic and delay aging, and on the other hand, they drive metabolic insufficiency to aggravate aging. Therefore, we need to find exosome and miRNA combinations that are more effective in delaying aging.

##### Soluble cytokine factors

Studies have shown that senescent hepatocytes secrete the paracrine senescence-related factor 13-Hydroxyoctadecadienoic acid, which acts on surrounding hepatocytes to promote hepatic steatosis.^188^ Importantly, senescent cells mediate the recruitment of neutrophils to the aging liver, which may be a mechanism by which aging spreads to surrounding cells.^189^ Fibroblast growth factor 21 (FGF21) is a longevity hormone secreted by the liver that is also produced in the thymic stroma. Mendelian randomization analysis also revealed that FGF21 has a beneficial relationship with potential genetic factors associated with aging.^190^ Paracrine FGF21 in the liver can increase liver regeneration and antioxidant capacity and inhibit liver aging^191^ and metabolic dysfunction-associated steatohepatitis.^192^ Similarly, the production of thymic FGF21 decreases with age.^193^ Thymic epithelial cells (TECs) constitute the main cell population involved in thymic aging.^194^ Thymic FGF21 overexpression increased TECs and decreased fibroblasts, and the regulation of TECs by FGF21 is required for thymic lymphopoiesis.^193,194^ In addition, BACH1 triggers FGF21 secretion by promoting ferroptosis,^195^ which in turn attenuates aging-related phenotypes, including cell senescence, obesity, and a shortened lifespan. FGF21 secretion is also required for lifespan extension by a protein-restricted diet^196^ or gut microbiota transplantation.^197^ In conclusion, paracrine FGF21 can delay aging through multiple pathways. Platelet-secreted chemokine platelet factor 4 (PF4/CXCL4) improves age-related cognitive impairment through hippocampal neurogenesis, which is the key to delaying the aging of the brain.^198,199^ In conclusion, intercellular communication plays a role as a “signal amplifier” during aging.

#### Chronic inflammation

The intricate interplay between chronic inflammation and the aging process has been the subject of extensive scientific inquiry, with this phenomenon formally termed “inflammaging”. Characterized as a low-grade, systemic chronic inflammatory state that progressively accompanies the aging process, inflammaging represents a distinct pathophysiological condition.^200^ Unlike acute inflammatory responses triggered by infection or injury, this persistent inflammatory phenotype arises from the dynamic interplay of multiple factors, including long-term immune system dysregulation, cellular stress responses, and metabolic derangements. This complex etiology underscores the multifactorial nature of age-related inflammatory processes and their critical role in mediating organismal senescence.

Multiple proinflammatory factors secreted by the SASP have been shown to play key roles in driving aging. IL-11 is a proinflammatory cytokine that plays a crucial role in maintaining human health. Under normal circumstances, IL-11 participates in regulating immune responses, but excessive levels can trigger unnecessary inflammation. As individuals age, the expression of IL-11 increases in cells and tissues, leading to fat accumulation and muscle loss. Anti-IL-11 therapy has been shown to prolong the average lifespan of male and female mice by 22.4% and 25%, respectively. In addition, neutralizing IL-11 antibodies combined with angiotensin-converting enzyme inhibitors synergistically prolong the lifespan of Alport syndrome mice.^201^ Dysregulation of IL-17 signaling disrupts epidermal homeostasis through activation of the NF-κB pathway, concurrently exacerbating systemic inflammatory cascades.^202^ Targeted suppression of IL-17 signaling in aging models attenuates cutaneous inflammation and delays the progression of age-associated phenotypes.^202^ Intra-articular injection of an anti-IL-17 neutralizing antibody reduced the expression of *Cdkn1a*, a marker of joint degeneration and aging.^203^ IL-6 signaling can drive cellular senescence through activation of the cGAS-STING-NF-κB pathway.^204^ In age-related cardiovascular disorders, IL-6 exacerbates mitochondrial dysfunction in vascular endothelial cells, thereby accelerating atherosclerosis progression via oxidative stress and lipid accumulation.^205^ Notably, the inhibition of IL-6 signaling ameliorates senescence-associated phenotypes in both Hutchinson-Gilford progeria syndrome-derived fibroblasts and progeroid mouse models, highlighting its therapeutic potential in counteracting premature aging.^206^ In addition, tissue-specific aging processes manifest distinct inflammaging signatures, with IL-17 predominating in cutaneous aging, IL-6 predominating in vascular aging, and TNF-α predominating in immune organ senescence.

The molecular mechanism of chronic inflammation-driven aging involves intricate crosstalk among multiple signaling axes within a distinct pathophysiological context. Recent advancements highlight the central roles of several evolutionarily conserved pathways in inflammaging. In addition to the previously discussed nutrient-sensing and autophagy pathways, the molecular landscape of inflammaging further encompasses several key regulatory signaling pathways: the cGAS-STING, Janus kinase-signal transducer and activator of transcription (JAK-STAT) pathways, Wnt/β-catenin signaling, and Notch signaling.

##### cGAS-STING pathway in aging

**Fig. 9:**
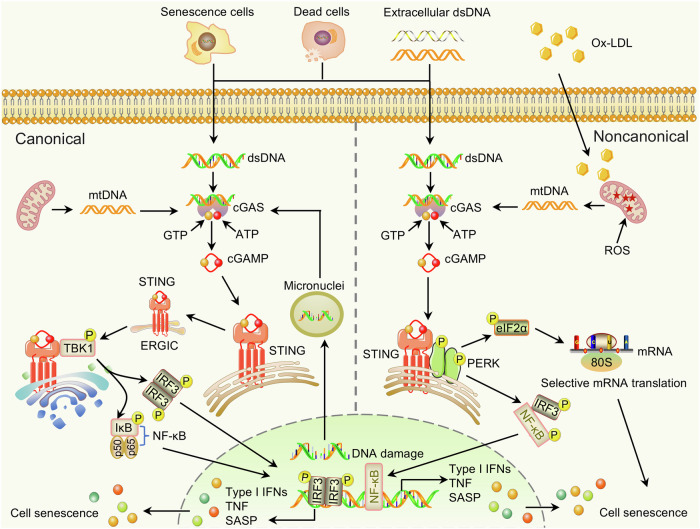
The cGAS-STING pathway in aging. In the canonical cGAS-STING pathway, abnormal dsDNA in the cytoplasm stimulates cGAS, causing conformational changes in cGAS, catalyzing the production of cGAMP from GTP and ATP substrates. cGAMP combines with STING dimers in the ER, resulting in conformational changes. STING translocates from the ER to the Golgi, where it recruits and activates TBK1 to phosphorylate IRF3. Subsequently, phosphorylated IRF3 forms dimers and transfers to the nucleus, inducing the expression of IFN-I. STING can also activate IKK to phosphorylate NF-κB, and phosphorylated NF-κB stimulates the expression of aging-related cytokines and proteins. Furthermore, the noncanonical STING-PERK-eIF2α pathway accelerates the synthesis of aging-related proteins. Some elements are derived from BioGDP (https://biogdp.com/)

cGAS-STING is an important pathway through which mammalian cells detect exogenous DNA and trigger innate immune responses. Since cGAS was first reported in 2013,^207^ the functions and key regulatory factors of the cGAS-STING pathway have been elucidated (Fig. 9). Accumulating evidence indicates that the cGAS-STING pathway accelerates SASP secretion via the aggregation of cytoplasmic DNA during senescence.^208,209^ Notably, cGAS-STING is related to multiple age-related diseases, including aging-related inflammation and neurodegeneration.^210,211^ Therefore, inhibition of the cGAS-STING pathway could decrease the SASP and delays cell senescence. The long interspersed nuclear element-1 (*LINE-1*) RNA retrotransposable element accumulates in progeria cells and inhibits the activation of the histone-lysine N-methyltransferase SUV39H1, ultimately resulting in heterochromatin loss and an aging phenotype.^212^ The cytoplasmic *LINE-1* cDNA stimulates the cGAS-STING pathway, promoting IFN-I and SASP responses.^213,214^ The process is antagonized by inhibitors (lamivudine) of the *LINE-1* reverse transcriptase. Importantly, knockout of LINE-1 RNA decreases the level of aging-related genes, thereby ameliorating age-related disorders.^212^ Remarkably, the noncanonical cGAS-STING-PERK-eIF2α pathway has been demonstrated to be critical for senescence and organ fibrosis.^215^ The activation of the cGAS-STING-PERK-eIF2α pathway remarkably inhibits mRNA translation but characteristically enhances the synthesis of longevity-related genes. Interference with the noncanonical cGAS-STING pathway through immunological, genetic, and pharmacological methods can notably ameliorate the cell senescence caused by lung and kidney fibrosis.^215^ In conclusion, suppression of the cGAS-STING pathway can be used to delay cell senescence and aging-related diseases.

At present, research on cGAS-STING pathway inhibitors has focused mainly on the targeted inhibition of the chief regulators cGAS, STING, and TBK1. Unfortunately, relatively few studies have investigated the pharmacological inhibition of cGAS-STING to delay aging. There is an urgent need to discover efficient and stable inhibitors as lead molecules. High-throughput screening and computer virtual screening can accelerate the discovery of cGAS-STING inhibitors, which is worthy of further research.

##### JAK-STAT pathway in aging

**Fig. 10:**
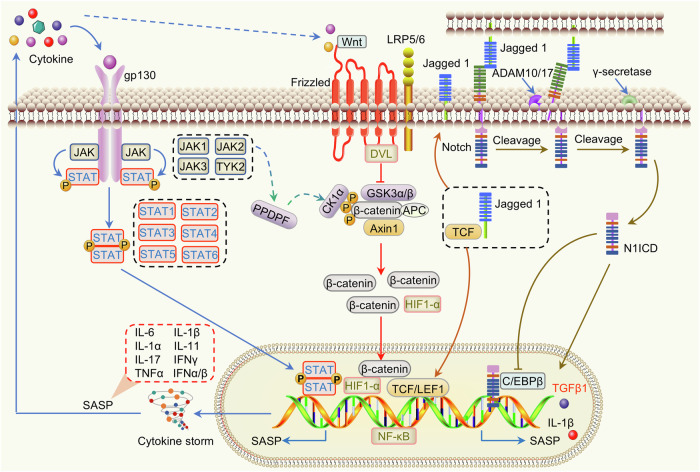
Crosstalk of the JAK-STAT, Wnt/β-catenin, and Notch signaling pathways in inflammatory aging. Cytokines specifically bind to membrane receptors, triggering a conformational change in the receptor and leading to dimerization and recruitment of relevant JAKs, thereby promoting JAK activation. Then, activated JAKs mediate tyrosine phosphorylation of STATs, and the phosphorylated STATs are separated from the receptor to form homodimers or heterodimers, which expose their nuclear localization signals and bind to the regulatory sequences of specific genes in the nucleus to regulate the expression of related genes. Senescent cells secrete many inflammatory factors (such as IL-6, IL-1β, and TNF-α), which continuously activate the JAK-STAT pathway. This continuous activation induces a cytokine storm, generating a large amount of inflammatory factors, which further exacerbates cellular senescence. Inflammatory conditions promote PPDPF phosphorylation via JAK2, and PPDPF activates the Wnt/β-catenin pathway by binding to CK1α. Wnt binds to receptors on the cell membrane (e.g., Frizzled, LRP) and inhibits the β-catenin degradation complex APC/GSK-3β/AXIN, allowing β-catenin to accumulate and enter the nucleus to regulate the expression of target genes (e.g., c-Myc and Cyclin D1). NOTCH1 can induce senescence through two different pathways, promoting TGF-β1-dependent secretomes and the proinflammatory cytokine SASP. The NOTCH1/TGF-β-regulated signaling network induces adjacent cell senescence in an autocrine/paracrine manner through Jagged 1 ligand/NOTCH1 receptor cell-cell interactions. On the other hand, N1ICD inhibits C/EBPβ-driven major proinflammatory secreted proteins

The JAK-STAT signaling pathway is a highly conserved signal transduction pathway that is involved in many human diseases, including cancer, autoimmune diseases, inflammation and blood diseases (Fig. 10). The JAK-STAT pathway, as the hub of cytokine signaling, is continuously activated in aging-related chronic inflammation. It drives multiple-organ functional decline by regulating inflammatory factor secretion, epigenetic remodeling and tissue-specific pathological processes.^216^ Senescent cells secrete SASP factors, which include many inflammatory factors (such as IL-6, IL-1β, and TNF-α) and growth factors. These factors act as “priming signals” that bind to receptors on the surface of neighboring cells to activate the JAK-STAT pathway. After JAK kinase is activated, it phosphorylates and dimerizes STAT proteins enters the nucleus, and binds to the regulatory region of specific genes, promoting the transcription of SASP-related genes (such as IL-6 and IL-8) by forming a vicious cycle of “inflammatory factor-activation of the JAK-STAT pathway-inflammatory factor storm”.^217^ This sustained JAK-STAT pathway activation puts the whole body in a state of chronic inflammation, accelerating vascular aging, tissue fibrosis,^218^ and metabolic disorders.^219^ Hence, JAK inhibitors have shown significant clinical advantages in reducing inflammation. The JAK-STAT signaling pathway has emerged as a central regulatory hub in the aging process by driving chronic inflammation, oxidative stress, and immunosenescence. Targeting this pathway is expected to disrupt the cycle and provide a new strategy for delaying aging and treating aging-related diseases. While inhibitors targeting this pathway, such as ruxolitinib, have demonstrated promise in preclinical models, their tissue specificity, long-term safety, and dynamic modulation requirements remain to be thoroughly explored.

##### Wnt-β-catenin signaling

The Wnt/β-catenin signaling pathway is a key pathway that regulates embryonic development, tissue homeostasis and stem cell self-renewal. Its abnormal activation or suppression is strongly linked to various diseases associated with aging (Fig. 10). During the aging process, oxidative stress, chronic inflammation and epigenetic alterations can lead to Wnt pathway dysregulation. Activation of the Wnt-β-catenin signaling pathway can promote the interaction between β-catenin and HIF-1α, impair mitochondrial function, and induce a glycolysis-dependent proinflammatory response.^220^ Moreover, chronic activation of Wnt-β-catenin induces considerable cellular senescence and promotes a fibrotic state. Wnt-β-catenin signaling is upregulated in aging kidneys^221^ and promotes aging-associated renal fibrosis by stimulating M2-type polarization of macrophages in a mouse model.^222^ In contrast, the activation of the Wnt-β-catenin pathway shows great potential in increasing osteogenic activity and inhibiting bone loss.^223^ Sclerostin is a bone factor secreted by mature osteocytes that regulates extraskeletal organ metabolism. Serum sclerostin gradually increases with age and can be used as a marker of aging. By binding to LRP5-6 receptors, sclerostin inhibits the Wnt-β-catenin pathway, which is critical for bone metabolism.^224^ In the pathogenesis of AD, the abnormal elevation of osteocyte-derived sclerostin may lead to cognitive impairment by inhibiting Wnt-β-catenin signaling in neurons through bone-brain crosstalk.^225^

The Wnt-β-catenin pathway plays a double-edged sword role in aging-related diseases by regulating stem cell function, the inflammatory response and tissue remodeling. In the process of aging, the Wnt-β-catenin pathway shows insufficient activity or overactivation in different tissues due to an imbalance of ligands and abnormal functions of key proteins, which drive age-related neurodegenerative diseases, cardiovascular diseases, and metabolic diseases, among others. Although therapies targeting this pathway have shown potential in preclinical and early clinical trials, their tissue-specific effects and long-term safety still need to be further explored.

##### Notch signaling

In the process of aging, the Notch signaling pathway is activated or inhibited in different tissues due to ligand-receptor imbalance, γ-Secretase abnormalities and downstream transcription disorders, which drive neurodegenerative diseases, cardiovascular diseases, immune senescence and metabolic disorders. Notch signaling is also elevated in human bone during aging,^226^ and loss of Notch signaling increases the number of skeletal stem and progenitor cells and improves the skeletal aging phenotype.^226^ miR-146a can counteract aging-related osteoarthritis by directly inhibiting the expression of IL-1β and IL6 mediated by the Notch signaling pathway.^227^ Endothelial Notch activation induces senescence and SASP in liver sinusoidal endothelial cells, which disrupts liver regeneration.^228^ γ-Secretase inhibitors in the Notch signaling pathway can inhibit senescence and promote liver sinusoidal endothelial cell expansion.^228^ SIRT1 is a direct target of Notch-Hes1, and Notch drives senescence of liver sinusoidal endothelial cells in a SIRT1-dependent manner. Activation of SIRT1 can neutralize the overexpression of p16, p21, and p53 generated through Notch activation.^228^ N1ICD, the intracellular domain of NOTCH1, inhibits the proinflammatory SASP by inhibiting C/EBPβ.^229^ Notch signaling regulates the stemness maintenance of hMSCs. The activation of the Notch signaling pathway mediated by its ligand Jagged 1 significantly inhibits hMSC senescence and promotes bone repair.^230^ Furthermore, Notch signaling suppresses senescence-associated heterochromatic foci and increases chromatin accessibility.^231^ Given the two distinct outcomes of Notch signal transduction, characterizing the properties of Notch signal transduction, including its broad functional roles, dependence on upstream and downstream signaling factors, precise spatiotemporal specificity, and crosstalk with other pathways, is essential. These characteristics may be responsible for the unclear mechanism by which Notch signaling regulates inflammatory aging-related diseases and the conflicting results produced by different research labs.

Proinflammatory cytokines serve as pivotal drivers of aging by initiating dysregulated inflammatory cascades, disrupting immune homeostasis, and triggering metabolic dysfunction, with their pathological effects manifesting across multiple biological scales, from subcellular processes to systemic pathophysiology. While current therapeutic strategies have demonstrated preliminary success in modulating these pathways, the field continues to grapple with fundamental challenges in maintaining equilibrium between inflammation control and host defense mechanisms while simultaneously advancing precision medicine approaches tailored to individual patient profiles.

#### Dysbiosis

**Fig. 11:**
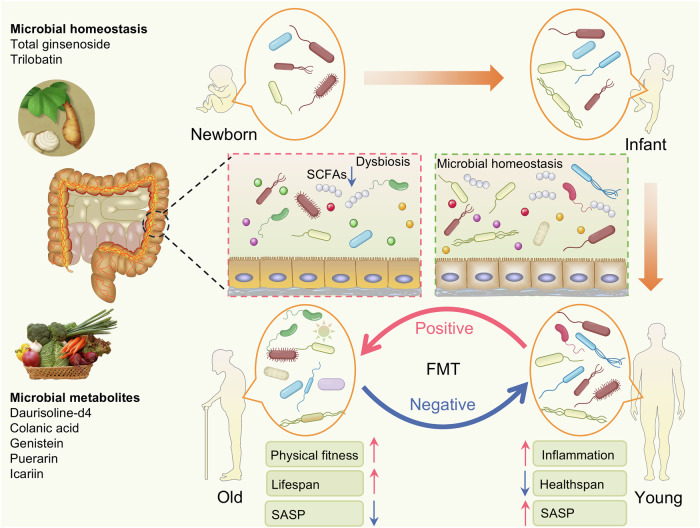
Regulation of dysbiosis to delay aging. In the gut of elderly individuals, the abundance of harmful flora increases. FMT can reverse the characteristics of multitissue aging by inhibiting inflammation. An increase in the level of microbiota-derived SCFAs regulates the homeostasis of the aging gut and extends the healthy lifespan. Natural products delay aging by regulating microbial metabolism and microbial homeostasis. Some elements are derived from BioGDP (https://biogdp.com/)

There is an inseparable relationship between the gut microbiota and host health. Dysbiosis promotes multiple pathological conditions, such as obesity, ulcerative colitis, nervous system diseases and cardiovascular diseases.^39,232^ The gut microbiota changes with age, and this change in the microbiota is closely related to age-related cognitive decline. Screening of 3983 *Escherichia coli* mutants revealed that deletion of 29 bacterial genes extends the lifespan of the host *C. elegans*.^233^ Colanic acid (capsular isopolysaccharide acid), a common gut bacterial metabolite extracted from five bacterial mutants, has been confirmed to extend the lifespan of nematodes and *Drosophila* by regulating ER stress and mitochondrial dynamics in host cells.^233^ Transplanting the gut microbiota of elderly mice into young mice can lead to loss of intestinal wall integrity in young mice, thereby promoting the release of microbiota metabolites into the circulatory system, leading to inflammation of the brain and eyes.^234^ Conversely, transplanting the gut microbiota of young mice into elderly mice can reverse the aging hallmarks of the gut, eyes, and brain.^234,235^ Additionally, fecal microbiota transplantation (FMT) from young mice restores the vitality of aging HSCs by inhibiting inflammation^236^ and regulating the availability of iron in the bone marrow.^237^ Supplementation of *Bifidobacterium longum* 68S mediates gut homeostasis, thereby exerting a delaying effect on skin aging,^238^ and improving age-related delays in fracture repair.^239^ Human progeria patients also exhibit gut dysbiosis, showing a remarkable increase in *Verrucomicrobia* and a decline in *Proteobacteria* in centenarians.^232^ Compared with older people, centenarians have a unique gut microbiota with special metabolites that can inhibit the growth of intestinal pathogens and reduce inflammation.^240,241^ Therefore, targeting the gut microbiota can provide means to delay aging (Fig. 11).

Numerous studies have shown that gut microbiota can be regulated by bioactive compounds through multiple pathways. They can directly suppress or stimulate the growth of certain bacteria, change the composition of the gut microbiota, and restore the balanced gut microbiota, thereby affecting host health. Secondly, they can cause changes in the content of bioactive microbial metabolites. Third, they regulate the secretion of substances by the gut microbiota, sustain the integrity of the intestinal mucosal barrier structure, and indirectly change the diversity of the intestinal microbiota. A variety of compounds, such as daurisoline-d4,^242^ trilobatin,^243^ genistein,^244^ puerarin,^245^ and icariin,^246^ have been shown to regulate intestinal homeostasis and delay aging.

### Emerging hallmarks of aging

In addition to the aforementioned aging hallmarks, novel aging hallmarks are being discovered. We summarized the newly discovered hallmarks of aging in Fig. 2d. They mainly include immunoglobin-associated senescence,^22^ endogenous retrovirus resuscitation,^45^ and centromere inactivation.^247^

#### Immunoglobin-associated senescence

Immunoglobulin G (IgG), the most important type of antibody in the body, has long been considered to be involved primarily in immune defense. However, in recent years, multiple studies have shown that IgG accumulates abnormally during the aging process and become an important factor driving tissue degeneration and metabolic decline. The accumulation of IgG is positively correlated with aging and is conserved in multiple species (including humans and mice),^22^ suggesting that it may serve as a cross-species hallmark of aging. Through the construction of a gerontological atlas of multiorgan aging, it was found that the accumulation of IgG is closely related to the increase in structural entropy and the loss of cell identity.^22^ During the aging process in mice and humans, IgG accumulates significantly in multiple organs, especially in metabolic- and immune-related organs such as white adipose tissue, the liver, and the spleen.^248^ For example, the IgG level in the white adipose tissue of elderly mice is nearly 20-fold higher than that of young mice.^248^ In addition, IgG can promote the senescence phenotype of macrophages and microglia, release inflammatory factors such as IL-6 and TNF-α, form a systemic chronic inflammatory environment, and accelerate multiorgan aging.^22^ Interference with IgG accumulation can significantly ameliorate metabolic decline during aging and prolong the healthy lifespan. The intervention strategy on the basis of antisense oligonucleotides reduces the IgG content in mouse tissues and delays multiorgan aging.^249^ In addition, caloric restriction has also been shown to delay aging by reducing IgG levels.^248^

In summary, the abnormal accumulation of IgG reveals the central role of the immunometabolic network during aging. The accumulation of IgG promotes systemic inflammation by activating macrophages, which can be attributed to the integrative characteristics of chronic inflammation. Future studies need to clarify the dynamic changes in IgG in different organs and develop therapies to precisely regulate IgG levels. Moreover, by combining spatiotemporal omics and big data models, the interaction between IgG and other aging hallmarks (such as chronic inflammation and stem cell depletion) can be analyzed to provide systematic solutions for delaying aging.

#### Endogenous retrovirus reactivation

Endogenous retroviruses (ERVs) are the remnants of ancient retroviruses integrated into the host genome, accounting for approximately 8% of the human genome.^45^ Recent studies have shown that the abnormal activation of ERVs is closely related to the aging process, which accelerates the occurrence of aging and related diseases through epigenetic dysregulation, the inflammatory response and intercellular signaling.^250,251^ Liu’s group systematically defined and revealed that aging-induced resurrection of endogenous retrovirus can serve as a hallmark of senescence.^40^ During aging, epigenetic regulation (e.g., decreased heterochromatin and disturbed DNA methylation) leads to the activation of otherwise silent ERV elements, the production of viral RNA and proteins, and packaging into viral particles. Their reverse transcription products (such as cDNA) trigger innate immune responses by activating the cGAS-STING pathway, leading to cell senescence and inflammation.^41,252^ At the same time, viral particles infect neighboring young cells through paracrine or humoral diffusion, spreading the senescence phenotype to the whole body through the mechanism of “contagious aging”, resulting in a cascade of amplification effects.^41^ Consistently, multiple high-throughput omics methods have revealed that the reactivation of ERVs directly leads to hair regeneration disorders and depletion of follicle stem cells.^253^ Notably, nucleoside reverse transcriptase inhibitors (abacavir and NRTIs) can alleviate age-related cellular and molecular changes.^252,253^ Intervention strategies against ERV provide novel approaches for delaying aging and preventing geriatric diseases. However, its biological complexity and clinical safety still need to be further explored. So does molecular mechanisms of ERV–host interactions.

#### Centromere inactivation

Centromeres are key structures on chromosomes responsible for chromosome segregation, and their dysfunction is closely related to cell senescence. Centromeric inactivation during aging can lead to chromosome segregation defects, genomic instability and decreased cell proliferation, which has become an important driver of aging.^247^ In senescent cells, the interaction between centromere protein B and lysine-specific demethylase-1 is increased, inhibiting H3K4me2 enrichment and thus hindering centromeric transcription. The centromere signature histone variant CENP-A and the structural protein CENP-C are significantly reduced.^247^ This decrease is directly related to abnormal chromosome segregation and increased micronucleus formation, suggesting that inactivation of centromere function is the core cause of mitotic defects in senescent cells. Chromosome missegregation caused by centromere inactivation leads to formation of micronuclei, activation of the cGAS-STING pathway, induction of chronic inflammation (such as the release of IL-6 and TNF-α), and formation of a pro-aging microenvironment.^247^ In addition, p53 protein levels are elevated in senescent cells and exacerbate centromere inactivation through the negative regulation of CENP-A expression.^247^ Caloric restriction or its mimetics such as rapamycin can indirectly maintain centromere function by regulating the mTOR pathway. In summary, centromere inactivation accelerates aging through epigenetic disorders, inflammatory cascades, and genomic instability. Intervention strategies targeting centromere inactivation have shown potential in cell models, and in the future, they need to be combined with multiomics analysis and clinical validation to promote the precise development of prolongevity treatments.

Collectively, the advances summarized above for the different hallmarks of aging highlight the importance of understanding the molecular mechanisms by which each hallmark influences aging pathologies and, more importantly, the functional interconnections between different hallmarks. Such an understanding forms the foundation for developing more effective and safer therapeutic interventions to combat these age-associated chronic diseases.

## Regulatory mechanisms of aging in aging-associated diseases

The hallmarks of aging and aging-related diseases do not exist in isolation. The hallmarks of aging present a series of characteristic changes at the molecular, cellular, and physiological levels during the aging process. These changes are not only reflected by the hallmarks of aging but also the core mechanisms driving the occurrence and development of aging-related diseases. The hallmarks of aging are the “upstream driving factors” of diseases, and in turn, diseases can accelerate the accumulation of aging hallmarks. Although all aging hallmarks are involved to a certain extent, different diseases may be dominated by specific hallmarks. The loss of protein homeostasis and mitochondrial dysfunction are particularly prominent in neurodegenerative diseases; genomic instability and epigenetic changes are the core in cancer; and chronic inflammation, cellular senescence and dysregulation of nutrient sensing are the keys in cardiovascular diseases. In summary, these aging hallmarks do not exist in isolation but are interrelated, forming a network that collectively promotes the decline of organismal functions.

### Tumorigenesis

Senescence plays a complex and seemingly contradictory dual role in tumorigenesis and progression. On the one hand, it promotes the occurrence of tumors; on the other hand, it may also inhibit the progression of tumors. This dual effect is closely linked to the biological characteristics of senescent cells and their microenvironment changes.^254^ Importantly, cancer and aging share critical hallmarks, including chronic inflammation, genomic instability, dysbiosis, and epigenetic alterations. Chronic inflammation underpins tumor initiation and progression. Senescent cells within the tumor microenvironment secrete a cascade of proinflammatory factors that promote tumorigenesis by inducing epigenetic instability, increasing cell proliferation, and stimulating angiogenesis.^255^ Increased levels of inflammatory cytokines, including IL-6 and IL-1, are associated with an increased risk of lung cancer in aging women, suggesting that aging promotes the development of cancer to a certain extent.^256,257^ The SASP secreted by senescent cells can also influence adjacent cells, including promoting tumor cell migration and metastasis, as well as facilitating angiogenesis and lymphangiogenesis by recruiting specific macrophages.

Senescent cells are in a steady state of cell cycle arrest, which is a natural barrier to tumorigenesis. While senescence helps suppress tumor initiation in the early stages of disease, increasing evidence indicates that the prolonged presence of senescent tumor cells often promotes tumor progression, metastasis, and recurrence. Moreover, senescent immune escape in cancer cells is a key contributor to antitumor therapy resistance and tumor relapse.^258^ With aging, the function of the immune system gradually decreases, and the ability of immune cells to recognize and eliminate tumor cells weakens, thereby allowing cancer cells increased opportunities to evade immune surveillance.^259^ Age-induced immunosenescence primarily affects effector T cells and other immune cell types that are critical for tumor immunity. These changes may lead to heightened activation and infiltration of immunosuppressive cell populations in elderly individuals, representing a key driver of their increased susceptibility to cancer and metastasis.^260^ Owing to intracellular and extracellular microenvironmental alterations and immunosenescence-related immune evasion, aging organisms provide a permissive environment for malignant tumor development.^261^ Conversely, following curative treatment, cancer survivors face long-term toxicity, including accelerated aging. In fact, they have a greater likelihood of cancer recurrence and an increased risk of developing a broader spectrum of aging-related diseases over time.

A number of studies have confirmed that senescent cell depletion strategies can eliminate senescent tumor cells and inhibit tumor progression.^255,262^ The senolytic drug D + Q has shown superior antitumor efficacy in liver cancer and colon cancer.^263^ Senescent cell-based cancer therapies include the induction of senescence in tumor cells and the subsequent selective elimination of senescent cells. That is, tumor cell senescence is induced, followed by the use of senolytic agents to eliminate residual senescent cancer cells.^263,264^ For example, tryptanthrin, a natural product, inhibits glutathione S-transferase P1 activity and promotes primary senescence of hepatocellular carcinoma cells. Tryptanthrin enhances the sensitivity of senescent hepatoma cells to Senololytic (ABT263)-induced apoptosis.^265^ Pharmacological inhibitors of cyclin-dependent kinases (CDKs) 4 and 6 (CDK4/6i) inhibit cancer growth by inducing a senescence-like state.^266^ However, long-term treatment efficacy is still limited by the development of drug resistance. The combination of a CDK4/6 inhibitor and an exportin 1 inhibitor can synergistically induce senescence in hepatocellular carcinoma cells, and the senolytic drug ARV-825 can effectively eliminate these senescent tumor cells.^267^ Although these trials utilized combination therapy rather than sequential therapy, strategies combining senescence inducers (e.g., etoposide, olaparib, or paclitaxel) with senolytics (e.g., navitoclax) have demonstrated clinical benefits in studies.^254^

The “one-two punch” therapies that combine pro-aging with antiaging therapies might be a good option for cancer treatment. However, there are still many challenges for this therapy, such as the development of drugs that induce cancer cell senescence selectively and efficiently as well as senolytic drugs with broad-spectrum effects.^268^ Additionally, the balance between inducing senescence and administering senolytic drugs should be controlled in terms of time and therapeutic efficacy.

### Hematopoietic system diseases

Hematopoietic aging is a pivotal driver of age-related anemia, clonal hematopoiesis, impaired adaptive immunity, and increased incidence of hematological malignancies in the aging population.

HSCs are the “seed cells” of the hematopoietic system, and their senescence is the core of hematopoietic system aging. Studies have shown that the function of aged HSCs decreases significantly, but the absolute number of HSCs increases instead (especially myeloid-biased HSCs). This increase in quantity may be a compensatory mechanism to compensate for functional deficits.^269^ The multilineage differentiation potential of aged HSCs is impaired, showing a bias toward myeloid differentiation (erythroid, granulocytic, and megakaryocytic lineages), whereas the differentiation ability toward lymphoid lineages (T cells, B cells) is significantly reduced. This directly leads to a decrease in lymphocyte production in elderly individuals, which is a key reason for the decline in adaptive immune function. Targeted clearance of myeloid-biased HSCs in aged mice via antibody therapy can reverse immune system aging.^182^ CRISPR-Cas9 screening revealed that Clusterin is a key regulator of aging-related myeloid bias, and knockout of Clusterin diminishes biased differentiation and restores the balance of the hematopoietic system.^270^ In addition, aged HSCs accumulate gene mutations, and HSC clones carrying these mutations gradually dominate hematopoiesis owing to their proliferative advantages, forming clonal hematopoiesis, which is the most important precursor lesion of myeloid malignancies.^271^ Its incidence increases sharply with age. The increased genomic instability of aged HSCs, decreased DNA repair capacity and selective pressures (such as inflammation) jointly promote the acquisition, expansion and dominant growth of mutant clones.^271^ Moreover, the ability of HSCs to migrate to and successfully colonize the bone marrow niche is weakened, which affects the function and maintenance of HSCs.^272^ Senescent HSCs may secrete proinflammatory factors, chemokines, and proteases, which affect themselves and other cells, disrupting the homeostasis of the bone marrow microenvironment.^273^ Therefore, imbalances in the quantity and quality of HSCs, imbalances in lymphoid and myeloid differentiation, and genomic instability are the main characteristics of HSC aging, driving the aging of the blood system.

The colonization, survival and functional maintenance of HSCs are highly dependent on the specific microenvironment (hematopoietic niche) formed by bone marrow stromal cells, the extracellular matrix and secreted cytokines. During aging, the structure and function of the hematopoietic niche are significantly altered, along with the abnormal composition of stromal cells, leading to a weakened ability to support HSCs.^274^ The secretion of CXCL12 (chemokine of HSCs) by senescent bone marrow endothelial cells is decreased, which also affects the colonization and survival of HSCs. In addition, the levels of proinflammatory factors (such as IL-6, TNF-α, and IL-1β) are increased in the aged hematopoietic niche, and these factors can disrupt the differentiation bias and functional decline of HSCs.^274^

In summary, HSC dysfunction, dysregulation of the hematopoietic microenvironment, and systemic inflammation constitute the core mechanisms of hematopoietic aging and jointly drive age-related hematological diseases.

### Neurodegenerative diseases

#### Alzheimer’s Disease

AD is the most common progressive neurodegenerative disorder characterized by cognitive decline and memory loss, and it primarily impacts the hippocampus and cerebral cortex.^275^ A key hallmark of AD is disrupted proteostasis, which leads to the pathological buildup of misfolded and aggregated proteins. These include amyloid-beta (Aβ) plaques, hyperphosphorylated tau tangles and the transactive response DNA-binding protein of 43 kDa (TDP-43).^276^ While AD has genetic causes, notably the apolipoprotein E4 (ApoE4) allele, which promotes the accumulation of Aβ plaques through the MAPK signaling pathway,^277^ the majority of cases are sporadic and strongly age-associated.^278^ A subaging population consisting of postmenopausal women can experience a sudden decline in estrogen levels, causing the brain to switch from glucose metabolism to lipid metabolism via white matter. This heightens women’s susceptibility to neuronal damage, leading to a higher (than men) incidence of AD as they age.^279^ Furthermore, aging can drive oxidative stress,^280^ DNA damage,^281^ epigenetic modifications,^282^ and mitochondrial dysfunction,^283^ which can also contribute to the accumulation of Aβ plaques and tau tangles. This buildup subsequently disrupts proteostasis and autophagy, ultimately hindering the removal of these toxic protein aggregates.

#### Parkinson’s Disease

PD, the most prevalent neurodegenerative disorder after AD, is a movement disorder characterized by the accumulation of α-synuclein aggregates,^284^ known as Lewy bodies, and impaired dopamine neurotransmission in the substantia nigra pars compacta,^285^ a region that controls movement. Like AD, PD is influenced by several genetic risk factors. Mutations at loci hinder lysosomal and mitochondrial function and obstruct protein clearance, increasing the accumulation of toxic protein aggregates and driving neurodegeneration. Aging accelerates the progression of PD through various mechanisms. Faulty proteolysis and hindered autophagy^286^ lead to the accumulation of α-syn aggregates, which can trigger disease progression once they exceed a specific threshold. Dysfunction of the PINK1-Parkin pathway in PD can lead to mitochondrial issues by obstructing mitophagy and disrupting ATP metabolism. As a result, the high-energy needs of dopaminergic neurons may be unmet, increasing their vulnerability to oxidative stress, which drives neurodegeneration. Additionally, chronic inflammation due to aging can compromise the immune system, leading to extended activation of glial cells.^287^ This ongoing activation initiates a detrimental cycle of inflammation in the brain, as these activated glial cells produce increased amounts of inflammatory cytokines. Ultimately, reducing or delaying these risk factors of aging may provide neuroprotection and delay the onset of disease progression.

#### Amyotrophic lateral sclerosis

Amyotrophic lateral sclerosis (ALS) is a swiftly advancing, fatal neurodegenerative disorder linked to mitochondrial dysfunction,^288^ primarily resulting in the degeneration of motor neurons within the central nervous system (CNS) because of unmet energy demands, leading to muscle weakness and eventually respiratory failure. Approximately 90% of ALS cases are sporadic, although mutations in some genes related to mitochondrial functions, such as HuD, TDP-43, and FUS, can increase the risk of disease progression.^289^ The incidence of ALS increases with age, peaking at approximately 70 years. In ALS, mutated TDP-43 can disrupt nuclear integrity and hamper the nonhomologous end-joining (NHEJ) pathways crucial for repairing double-strand DNA breaks in neurons.^290^ With aging, mitochondrial dysfunction and genomic instability can lead to the buildup of oxidative stress and DNA damage, which can disrupt energy production and genomic maintenance in motor neurons, processes implicated in ALS pathogenesis. Moreover, ALS is often further characterized by dysregulated proteostasis and autophagy, which can cause an excess of unfolded proteins, including stress granules, and TDP-43 pathologies.^291^

#### Huntington’s Disease

HD is a hereditary neurodegenerative condition caused by a mutated huntingtin (HTT) gene on chromosome 4.^292^ In HD, the GABAergic neurons in the striatum and cortex are the most affected, contributing to clinical hallmarks such as chorea, cognitive impairment and nonmotor symptoms.^293^ Telomere attrition and increased DNA methylation with aging can accelerate the onset of HD.^294^ Inhibition of autophagy via TFEB activation can subsequently inhibit calcineurin activity, which is required for neuronal transmission.^295^ Excessive mutHTT can overwhelm the UPS, dysregulating proteostasis and further promoting mutHTT accumulation.^296^ The presence of mutHTT and the accumulation of mtDNA mutations caused by inadequate mitophagy and decreased biogenesis impair oxidative phosphorylation, enhance the formation of reactive ROS, and lower ATP production. This leads to inefficient energy generation and heightens the likelihood of neuronal degeneration.^297^ With HD, senescent neural cells and neurons can arise because mutHTT contributes to chronic neuroinflammation via the secretion of SASP.^297^

### Cardiovascular diseases

#### Atherosclerosis

Atherosclerosis is a chronic inflammatory disease characterized by the accumulation of plague made of cholesterol, fat, calcium, dead cell waste components, and immune cells in arterial walls. This can restrict blood flow, depriving vital organs of oxygen.^298^ It can trigger cardiovascular diseases such as heart attack and strokes. Key risk factors for atherosclerosis include hypertension, obesity, and maternal-fetal blood transfer.^299^ Poor diets or metabolic conditions can lead to independent atherosclerosis risk factors such as increased ceramide levels.^300^ Genetic mutations that affect inflammatory pathways, such as the activation of NF-κB, may increase the risk of atherosclerosis.^301^ Aging causes internal and external alterations in blood arteries, usually preceding atherosclerosis.^205^ Understanding these mechanisms may enable early interventions to lessen the effects of atherosclerosis. Telomere attrition in leukocytes can lead to genomic instability, initiating senescence in vascular smooth muscle cells (VSMCs) and subsequently promoting a proinflammatory environment. Aging may lead to diminished regeneration and functionality of VSMCs, potentially promoting the formation of atherosclerotic plaques and weakening the structural integrity of blood vessel walls.^302^ The age-related decrease in NRF2 expression may result in a loss of proteostasis and mitochondrial dysfunction. This leads to increased systemic ROS, which can contribute to cardiovascular events and promote atherosclerosis.^303^

#### Heart failure

Heart failure (HF) is a clinical illness characterized by structural impairment of the heart, impairing its capacity to deliver adequate blood to satisfy the metabolic requirements of essential organ functions. Left ventricular HF often results from coronary heart disease, hypertension, or myocardial infarction, whereas right ventricular HF is more commonly linked to pulmonary conditions such as chronic obstructive pulmonary disease (COPD). The role of aging in HF is significant. Aging can impair heart function, leading to disrupted proteostasis. This then drives oligomeric aggregation of proteins in the myocardium and reduced levels of extracellular clusterin, contributing to reduced ejection fraction.^304^ Elevated concentrations of insulin-like growth factor-binding protein-7 (IGFBP7) are recognized to facilitate cardiac senescence, hindering DNA repair and resulting in ROS production, thus expediting the onset of HF.^305^ The mitochondrial dysfunction associated with aging can impair the ability of cardiac muscle to fulfill ATP demands. It also disrupts calcium homeostasis, contributing to increased ROS, which can cause proteotoxic damage and lead to cardiomyocyte death. Ultimately, these factors create a vicious cycle that contributes to HF.^306^

#### Stroke

Stroke is a significant cerebrovascular incident that is generally categorized into two types: ischemic and hemorrhagic. Ischemic strokes account for approximately 80% of cases and occur due to blockage of blood flow caused by thrombosis in the vessels. In contrast, hemorrhagic strokes result from ruptured blood vessels caused by increased intracranial pressure due to tumors, injury, or inflammation. Genetic risk factors for ischemic stroke overlap with atrial fibrillation and coronary heart disease, whereas APOE and PMF1 are associated with hemorrhagic stroke.^307^ After reaching 55 years of age, an individual’s risk of stroke is expected to double every decade.^308^ This may be associated with aging hallmarks such as telomere shortening.^309^ A reduction in telomere length can drive cells into a senescent state, which triggers chronic inflammation through the NF-κB pathway. This inflammation can disrupt matrix integrity, resulting in cell fibrosis, endothelial damage, and increased oxidative stress. Moreover, this inflammatory state may lead to a potential loss of proteostasis, autophagy, and mitochondrial dysfunction,^310^ diminishing the ability of cells to repair or regenerate, further exacerbating the pathologies linked to stroke progression.

### Metabolic disorders

#### Insulin resistance and diabetes

Type 2 diabetes mellitus, metabolically associated fatty liver disease, neurodegenerative diseases, and cardiovascular diseases are significantly increased by insulin resistance.^311^ Global projections indicate that the prevalence of diabetes is expected to reach 11% by 2045, affecting 700 million people, whereas impaired glucose tolerance is anticipated to rise to 9%, impacting 548 million individuals worldwide.^312^ Moreover, age serves as a significant risk factor, with the incidence of diabetes doubling for those over 65 years and increasing fourfold for individuals above 75 years old.^313^ For instance, a study reported that gut bacteria from the Lachnospiraceae family contribute to insulin resistance by promoting lipid accumulation and chronic inflammation, whereas bacteria in the genus Bacteroides counter insulin resistance.^313^ As mitochondrial dysfunction increases with age, it can reduce ATP levels and increase oxidative stress in various tissues, including cardiac, hepatic, and pancreatic β-cells. This process can lead to their dysfunction and contribute to insulin resistance.^314^ Hormonal changes associated with aging, such as decreased estrogen during menopause, can increase insulin sensitivity in adipose tissue. As we age, decreasing adiponectin levels may worsen glucose sensitivity and lipid metabolism.^315^ Dysfunctional autophagy in adipose tissues can lead to ER stress and disrupt insulin signaling, contributing to insulin resistance in adipocytes.^316^

#### Bone metabolism dysregulation and osteoporosis

Maintaining bone mass requires a delicate balance between osteoblast-mediated bone production and osteoclast-mediated resorption. When this equilibrium is disrupted by excessive bone resorption or impaired bone formation, bones become more porous and brittle. Bone metabolism dysregulation worsens with age. Bone mass decreases steadily by approximately 10 g/Ca annually, with females experiencing accelerated loss postmenopause.^317^ With age, the accumulation of senescent bone marrow mesenchymal stem cells and macrophages (S-MΦs) can activate the secretion of SASP.^318^ SASP, such as TNF-α, IL-1, and IL-6, can increase osteoclast activity, accelerate bone resorption and contribute to decreased bone mineral density.^319^ Additionally, in aging osteoprogenitor cells, telomere attrition, DNA damage, and dysregulation of signaling pathways such as mTORC1 and Nrf2 reduce bone formation.^319^ Mitochondrial dysfunction in bone cells hinders energy generation, increases ROS, and disrupts the balance between osteoblasts and osteoclasts.^320^ These interconnected aging mechanisms undermine bone integrity, driving osteoporosis severity and complicating recovery.

#### Metabolic fatty liver disease

Metabolically associated fatty liver disease (MAFLD) encompasses a spectrum of liver diseases not covered by its previous terminology of nonalcoholic fatty liver disease^321^ MAFLD has become the leading cause of chronic liver disease, affecting approximately 25% of the world’s population as of 2024. Age and MAFLD incidence are correlated, with the median age of the MAFLD population being 50 years.^322^ Several aging-related hallmarks have been identified that exacerbate MAFLD, which will be discussed in more detail. Impaired MQC resulting from reduced mitochondrial biogenesis (decreased PGC-1α) and mitophagy (impaired PINK1/Parkin pathway) leads to oxidative stress and free fatty acid buildup in hepatocytes.^323^ Elevated IL-6 levels can reduce insulin signaling and contribute to lipid accumulation and hepatic fibrosis.^324^ Furthermore, as we age, gut dysbiosis may reduce the beneficial bacteria responsible for activating pancreatic lipase, which is essential for efficiently breaking down lipids and preventing their accumulation.^325^

#### Hyperuricemia and Gout

Hyperuricemia poses a worldwide health issue, impacting 3% to nearly 40% of individuals. Its prevalence is influenced by demographic factors and is linked to painful conditions such as gout and kidney stones.^326^ It can be further defined as a serum uric acid level exceeding 6.8 mg/dL (the saturation points for monosodium urate crystal formation). This condition primarily stems from renal underexcretion caused by dysfunctional urate transporters. However, the overproduction of uric acid caused by enzymatic abnormalities, such as deficiency of hypoxanthine-guanine phosphoribosyl transferase in nucleotide metabolism, may also contribute to elevated urate levels. Chronic hyperuricemia promotes the deposition of monosodium urate crystals in joints and renal tubules, leading to gout^327^ and kidney stones,^328^ respectively. This accumulation activates the innate immune response via NLRP3 inflammasome-mediated IL-1β production, further propagating inflammation.^329^ Kidney function diminishes with age, reducing uric acid excretion and increasing serum uric acid levels.^326^ Age-related immunosenescence and chronic kidney disease impair immunological clearance and renal regeneration, potentially leading to irreversible renal damage.^330^ Furthermore, gut dysbiosis, which involves bacteria that affect purine metabolism, can lead to inflammation and contribute to hyperuricemia and gout.^331^

### Autoimmune diseases

#### Rheumatoid arthritis

RA develops when the immune system targets healthy synovium, causing chronic inflammation and cartilage loss.^332^ Age affects genetic and environmental susceptibility by affecting immune regulation and metabolic homeostasis. The lifetime risk of developing RA ranges from 1 to 3%, peaking after the age of 50 years,^333^ with a marked increase in incidence among women.^334^ Aging is associated with elevated levels of leptin, an adipokine that accumulates in adipose tissue and is exacerbated by obesity.^335^ Leptin stimulates both the innate and adaptive immune systems, promoting the production of proinflammatory cytokines such as IL-1, IL-6, and TNF-α, contributing to systemic and synovial inflammation.^336^ As people age, their DNA repair mechanisms weaken, increasing susceptibility to infections. This can result in immune dysregulation and reduced self-tolerance. Consequently, rheumatoid factor and antinuclear antibodies, often found in autoimmune disorders such as RA, may be produced more readily owing to this immunological imbalance.^337^ Concurrently, age-related decreases in PINK1/Parkin-mediated mitophagy promote oxidative stress and mitochondrial dysfunction, which can trigger synovial inflammation and reduce the osteoclast activity critical for healthy bone remodeling, thereby contributing to joint damage and motor impairment.^338^ The accumulation of ROS further exacerbates cellular stress by inducing genotoxic damage, which can lead to overactivation of the p53 tumor suppressor gene, driving persistent senescence and amplifying inflammatory signaling through increased cytokine and metalloproteinase production, potentially accelerating the onset or progression of RA and other age-related inflammatory conditions.^339^

#### Multiple sclerosis

Multiple sclerosis is another autoimmune disease mediated by the activation of self-reactive T cells. While multiple sclerosis can affect children, late-onset multiple sclerosis can occur in individuals older than 50 years because of age-related changes.^340^ Research has shown that aging exacerbates multiple sclerosis pathology through several biological mechanisms, which span from increased neuroinflammation and a dysregulated immune system to impaired remyelination and gut dysbiosis.^341^ A population-based study revealed that late-onset multiple sclerosis most commonly occurs between the ages of 50 and 55, accounting for approximately 69% of late-onset multiple sclerosis cases.^342^ Immune senescence, particularly of cytotoxic CD4 + T cells,^343^ promotes chronic inflammation and the accumulation of somatic single-nucleotide variants in the CNS.^344^ These mutations can enhance neuroinflammatory cascades. Chronic inflammation leads to elevated nitric oxide production, which can cause axonal demyelination and downstream mitochondrial dysfunction.^345^ Dysfunctional mitochondria in demyelinated axons fail to meet the high ATP demands of Na/K-ATPases, thus impairing neurotransmission.^346^

#### Systemic lupus erythematosus (SLE)

The production of antinuclear antibodies is a hallmark of the chronic autoimmune disease known as SLE.^347^ SLE is a clinically heterogeneous disease in which patients experience manifestations ranging from mild cutaneous involvement, such as flashes, to life-threatening organ dysfunctions.^348^ SLE shares similar risk factors with the other autoimmune diseases mentioned above. These factors can trigger or worsen the autoimmune response when they cause immune dysregulation, impacting both the innate and adaptive immune systems. The accumulation of cellular debris and apoptotic cells may act as a source of autoantigens and cause inflammation. This triggers the adaptive immune response, which activates T cells to release additional inflammatory cytokines to activate B cells. The development of autoimmunity can be promoted by the capacity of these cells to generate specific autoantibodies to target self-antigens.^349^ According to epidemiological studies, the global burden of SLE varies considerably on the basis of demographic factors. Prevalence estimates range from 13 to over 7000 per 100,000 individuals, whereas incidence rates range from approximately 1.5 to 11 per 100,000 person–years. The prevalence of SLE peaked in the 60- to 64-year-old age range in women,^350^ suggesting that age may act as a risk factor for SLE. Chronic inflammation caused by senescent T cells can compromise proteostasis and lead to mitochondrial dysfunction, contributing to immunosenescence.^351^ Mitochondrial dysfunction is associated with triggering autoimmune responses because it releases mtDNA that can initiate IFN-I signaling.^352^

### Skin aging

The skin, the body’s largest organ, consists of the epidermis, dermis, and hypodermis. Intrinsic biological alterations, exacerbated by environmental stressors such as UV radiation, pollution, and smoking, can disrupt skin homeostasis with age. These cumulative insults lead to epidermal thinning, degradation of collagen and elastin, and impairment of the skin barrier, which manifest clinically as wrinkles, delayed wound healing, and increasing vulnerability to dermatoses.^353^ In a clinical cohort study of the Singaporean population, dermatitis was the most prevalent dermatosis across all age groups (33.3%), rising to 37.7% among the aged, underscoring how age is a key risk factor.^354^ Many age-associated dermatoses can develop or contribute to chronic skin disorders such as actinic keratosis (precancerous lesions), psoriasis,^355^ prurigo nodularis,^356^ and pigmentation issues such as senile lentigo and melasma.^357^ Exposure to UV light can lead to mtDNA mutations and the accumulation of dysfunctional mitochondria, disrupting collagen homeostasis and driving oxidative stress accumulation.^358^ As oxidative stress accumulates, DNA damage occurs, promoting the development of senescence in keratinocytes, melanocytes, and dermal fibroblasts. Senescence can disrupt skin intercellular communication and tissue structure.^357^ Accumulation of senescent cells drives ‘inflammaging’, further accelerating collagen degradation through TGF-β-Smad signaling.^359^

## Therapeutic strategies to counteract aging and aging-associated diseases

Aging-related diseases do not exist in isolation; their common essence lies in the cumulative damage caused by aging hallmarks. The mechanisms of aging are the common root cause of these diseases, and healthy longevity and antiaging therapies can achieve broad-spectrum prevention or improvement of various diseases by intervening on the “upstream mechanisms” of aging hallmarks, thereby fundamentally improving the environment for disease occurrence. Therefore, aging hallmarks serve as a controlling point, and antiaging therapy can achieve upstream control of multiple diseases by reducing the hallmark levels. This relationship provides a novel perspective for the prevention and treatment of complex diseases in the elderly population. There are multitude of findings in longevity research and in the following section, we discuss several of the most promising antiaging methods.

### Senolytics

Previous studies have confirmed that elimination of *p16*^*INK4A*^-positive senescent cells successfully delays aging-related diseases.^360^ Senolysis, which selectively kills senescent cells, was first proposed in 2015.^29^ The first senolytic agent is the D + Q combination,^29^ and the combination therapy of D + Q has been used in multiple aging-related studies in the clinical stage. The drug combination can selectively kill senescent cells; the average lifespan of treated elderly mice is prolonged by 36%, and mortality is reduced by 65%.^361^ Aging-associated osteoporosis and senescent cells are causally connected. Senolytics suppress osteoclast-mediated bone resorption and support the maintenance and activity of osteoblasts,^362^ ameliorate postmenopausal osteoporosis and rejuvenate bone regeneration.^363^ Moreover, D + Q eliminates senescent cells and accelerates fracture healing in elderly individuals.^364^ In neurodegenerative diseases, D + Q alleviates progenitor cell senescence and age-associated cognitive impairment.^365^ In conclusion, senolytics (D + Q) have strong efficacy in delaying age-related diseases. To improve the accuracy of clearing senescent cells, the senolytic drug dasatinib was fused with the lipofuscin-binding domain, a senescence marker, and embedded into the nanomicelles to form a novel drug system mGL392.^366^ Compared with D + Q, this drug has greater potential for the precise elimination of senescent cells.

**Fig. 12:**
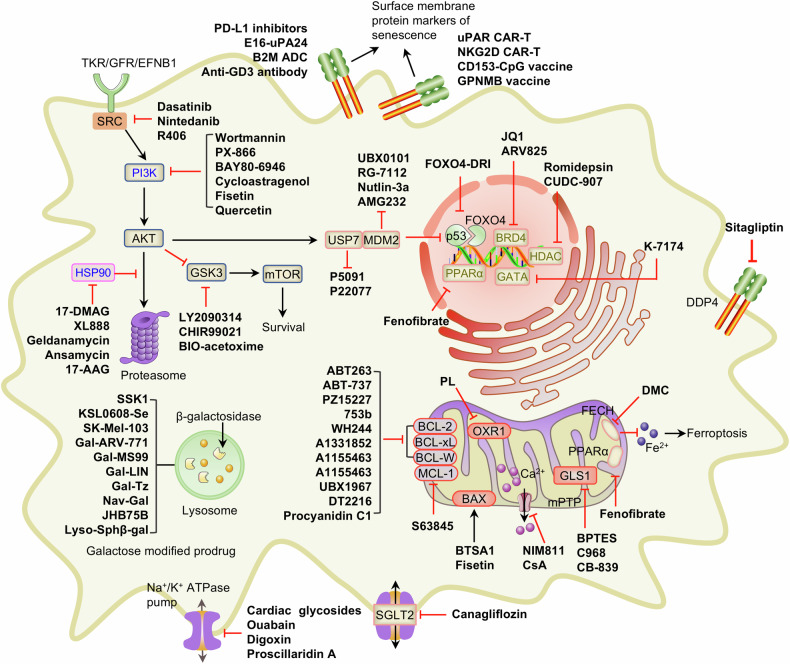
Senolytic agents and their targets. It depicts inhibitors that target key signaling cascades (SRC-PI3K-AKT-mTOR), aging-related epigenetic regulators, and organelle-specific effectors. Small molecule inhibitors, prodrugs, and immunotherapies (such as CAR-T cells and vaccines) that act on components, including cell surface markers, kinases, epigenetic regulators, and metabolic enzymes, have also been identified

Currently, small molecules known to target BCL-2/BCL-xL, BET family proteins, glutaminase 1 (GLS1), heat shock protein 90 (HSP90), tyrosine kinase or N-myristoyltransferase^367^ (Fig. 12, Table 2) can be used as senolytic agents. ABT263 (navitoclax), a Bcl-xL/Bcl2/Bcl-w inhibitor, was initially screened as a senolytic agent that could be used to eliminate senescent HSCs and hMSCs.^30^ Intraperitoneal injection of ABT263 significantly accelerated wound healing in diabetic mice by eliminating senescent cells.^368^ ABT263 has shown superior therapeutic effects in a variety of aging-related diseases by eliminating senescent cells, such as spinal pain,^369^ vascular aging,^370^ pulmonary hypertension,^371^ photoaging,^372^ and cancer.^373^ Unfortunately, platelet dose toxicity has been found to limit its clinical application. To increase the specificity of ABT263, the proteolysis-targeting chimera (PROTAC) platform was used for modification to generate three dual BCL-xL and BCL-2 inhibitors, PZ15227,^374^ 753b,^375^ and WH244.^376^ 753b can effectively clear chemotherapy-induced senescent acute myeloid leukemia cells.^375,377^ Importantly, 753b and PZ15227 can prevent the platelet toxicity caused by the first-generation Bcl-xL/Bcl-2 dual inhibitor navitoclax.^374^ Recent research has shown that 753b can selectively eliminate senescent liver cells and treat metabolic dysfunction-associated steatotic liver disease.

**Table 2:** Currently identified senolytic agents

Target types	Drugs	Highlights	Ref.
Tyrosine kinase inhibitor	Dasatinib	Clearing senescent cells alleviates a variety of aging-related diseases	^361,365,504^
	Nintedanib	Inhibition of STAT3 induces selective cell death in senescent cells	^505^
BCL-2 family proteins inhibitor	ABT263 (navitoclax)	Senolytic in HUVECs, IMR90, and MEFs, but not human primary preadipocytes	^29^
	ABT-737	Elimination of senescent cells induced by DNA damage in the lung	^506^
	PROTAC PZ15227	Effective elimination of senescent cells from naturally aging mice	^374^
	PROTAC 753b	Selective elimination of senescent cells in the liver of aged mice; eliminates leukemia cells	^31,375^
	PROTAC WH244	The degradation ability of BCL-xL/BCL-2 is better than that of 753b	^376^
	A1155463	inducing senescent myotonic dystrophy type 1 myoblasts apoptosis	^507^
	UBX1967	Clearing p16ink4a positive senescent cells and improving retinopathy	^508^
	PROTAC DT2216	Specifically targeted degradation of BCL-xL	^509^
Mcl-1 inhibitor	S63845	Eimination of senescent tumor cells and metastases	^262^
BAX activator	BTSA1	Selectively promoted the apoptosis of senescent myofibroblasts	^510^
DPP-4 inhibitor	Sitagliptin	Selective depletion of DPP-4 positive senescent chondrocytes	^511^
BET family proteins	PROTAC ARV825	Attenuation of nonhomologous end joining (NHEJ), and activation of autophagy	^267,512^
HDAC inhibitor	Romidepsin	Elimination of senescent immunosuppressive neutrophils in a TREM2-dependent manner	^513^
GATA inhibitor	K-7174	Interferes with the interaction of ZMIZ1-GATA4, eliminates senescent chondrocytes and improves osteoarthritis	^514^
SGLT2 inhibitor	Canagliflozin	Downregulation of PD-L1 expression enhances immune-mediated elimination of senescent cells	^515^
GSK3 inhibitor	LY2090314 CHIR99021 BIO-acetoxime	Selectively kill senescent liver cancer cells	^516^
cIAP2 inhibitor	Birinapant	Specific clearance of senescent glioblastomas cells	^517^
GLS1 inhibitor	BPTES C968 CB-839	Inhibition of glutaminolysis specifically abolished senescent cells, ameliorates kidney aging	^518^
PPARα agonist	Fenofibrate	Promotes apoptosis of senescent cells, increases autophagy flow, and prevents cartilage degradation	^519^
Casein kinase 2 inhibitor	4,5,6,7-tetrabromo-2-azabenzamidazole (TBB)	Preferentially trigger apoptosis in senescent Zmpste24-/- cells	^520^
Pan-PI3K inhibitor	Wortmannin PX-866 BAY80-6946	Efficiently and specifically eliminated senescent cells	^521^
PERK inhibitor	GSK2606414	Induces residual senescent apoptosis	^522^
Heat shock protein CRYAB inhibitor	25-hydroxycholesterol	Targeted inhibition of CRYAB	^523^
HSP90 inhibitor	17-DMAG Geldanamycin	Prolonging the healthy lifespan of Ercc1 -/∆ mice	^524^
	K4 and K5	Prolong the lifespan of *Drosophila* and reduce aging markers in aged mice	^525^
	XL888	Elimination of pathogenic p16Ink4a+ fibroblasts from mouse models of pulmonary fibrosis	^526^
N-myristoylation inhibitor	IMP1088 DDD86481	Inhibition of COPI selectively eliminates senescent cells and ameliorates cancer and NASH	^367^
FOXO4-p53 inhibitor	FOXO4-DRI peptide	Interfering with FOXO4-p53 interaction selectively induces p53 nuclear exclusion and endogenous apoptosis in senescent cells	^527^
MDM2-p53 inhibitor	UBX0101	Inhibiting p53/MDM2 interaction alleviates osteoarthritis	^452^
	RG-7112 and o-Vanillin	Reduced the amount of senescent intervertebral disc cells	^528^
	Nutlin-3a (Rebemadlin) AMG232 (Navtemadlin)	Reduced epigenetic age	^529^
USP7-MDM2-p55 inhibitor	P5091 P22077	USP7 inhibition promotes ubiquitination and degradation of MDM2, increases p53 levels, and selectively induces apoptosis in senescent cells.	^530^
Mitochondrial Integrity	Tamoxifen	Targeting mitochondria of senescent cells alleviates obesity and type 2 diabetes	^531^
Ca2+ communications (Cyclophilin D inhibitor)	NIM811 Cyclosporin A (CsA)	Pharmacological blockage of CypD leads to preferential elimination of senescent cells	^532^
Immune-mediated senescent cell clearance	PD-L1 inhibitors	Eliminate PD-L1 positive senescent cells	^533^
	E16-uPA24	Enhance the direct coupling of senescent cells and immune cells and induce immune clearance of senescent cells	^394^
	uPAR CAR-T	Effectively removes senescent cells and improves metabolic dysfunction both in vitro and in vivo	^35,395^
	NKG2D CAR-T	Targeted elimination of senescent cells	^397,398^
	CD153-CpG vaccine	Elimination of senescent T cells	^534^
	GPNMB vaccine	Attenuate senescence in adipose tissue and extend lifespan of progeroid mice	^535^
Natural products	Fisetin	Targeted elimination of senescent cells prolongs median and maximum lifespan	^29,389^
	Procyanidin C1	Exhibits senomorphic and senolytic functions, alleviating lung/kidney fibrosis and age-related retinal impairment, and prolonging lifespan	^390,391^
	Ginkgetin Periplocin Oleandrin	Screening by machine learning	^441^
	Luteolin Phloretin Baicalein 3,4’-dihydroxypropiophenone α, β-dehydrocurvularin	Screening by computer programs Chemminer and rcdk programs	^536^
	Cycloastragenol	Exhibits senomorphic and senolytic functions	^537^
	Flavonoid 4,4’-dimethoxychalcone (DMC)	Inhibited FECH triggering ferroptosis of senescent cells	^392^
	Oridonin	Targeting glutathione S-transferase, activates ROS-P38 signaling and induces apoptosis in senescent cells	^538^
	Pinosylvin	Potential senolytic agent for pulmonary fibrosis	^539^
	Cardiac glycosides (Ouabain, Digoxin, Proscillaridin A)	Inhibition of Na+/K+ ATPase, induction of Bcl-2 family protein NOXA mediates apoptosis of senescent cells	^393,540,541^
	Piperlongumine (PL)	Targeted OXR1 inducing senescent cells apoptosis	^542^
	Ligustilide	Exhibits senomorphic and senolytic functions	^543^
	JM10101	Exhibits senomorphic and senolytic functions	^544^
Galactose modified prodrug	SSK1	Eliminate senescent cells in different tissue	^379^
	KSL0608-Se	Mediated photodynamic therapy effectively eliminated senescent cells in the liver and kidney of naturally aging mice	^380^
	SK-Mel-103	Novel Senolytic agents based on photodynamic therapy	^381^
	PROTAC Gal-ARV-771 Gal-MS99	Galactose and PROTACs constitute prodrugs that enhance senolytic indexes	^382^
	Gal-LIN	Decreased SA-β-gal levels in MLN-induced senescent cells and induced apoptosis	^383^
	Gal-Tz	The prodrug of PROTAC, TCO-ARV-771, can be selectively activated by Gal-Tz to induce BRD4 degradation and subsequent apoptosis	^384^
	Nav-Gal	Selectively induced senescent apoptosis	^378^
	JHB75B	Elimination of chemotherapy-generated senescent cells and preneoplastic senescent cells	^545^
	Lyso-Sph_β-gal_	Targeting lysosomal metabolism defects to eliminate senescent cells	^546^
Nanomicelle	mGL392	Lipofuscin-binding domain scaffold conjugated with dasatinib,	^366^
	SSK1-NPs	SSK1-loaded neurotransmitter-derived lipid nanoparticles	^386^
	NPs(Nav)@MMP-3	Mesoporous silica nanoparticles (MSNs) were loaded with navitoclax and coated with a substrate-specific peptide of MMP-3	^387^
	MNPQ	Quercetin surface functionalized Fe3O4 nanoparticles	^547^
	(AspSerSer)_6_-sEVs DM1-Gal	Specifically eliminate senescent osteocytes	^548^

Designing prodrug molecules using senescence-associated β-galactosidase (SA-β-gal) has become a potential strategy to improve target specificity and reduce platelet toxicity.^378^ On the basis of the increased activity of β-gal in senescent cells, a new prodrug, SSK1, which can be specifically cleaved by lysosomal β-gal into cytotoxic substances and induce the apoptosis of senescent cells, thereby eliminating senescent cells, was developed.^379^ In recent years, a variety of senolytic agents have been developed on the basis of β-gal, such as the photosensitive senolytic prodrugs KSL0608-Se,^380^ SK-Mel-103,^381^ PROTACs Gal-ARV-771,^382^ Nav-Gal,^378^ Gal-LIN,^383^ and Gal-Tz,^384^ etc. To increase the specificity of senolytics, these agents are packaged into galactose micelles or nanovesicles to achieve specific delivery and reduce side effects.^385^ SSK1-loaded neurotransmitter-derived lipid nanoparticles, as novel nanomedicines, can penetrate the permeability of the blood-brain barrier, eliminate senescent cells, reduce the deposition of β-amyloid, and improve the cognitive function of aged AD mice.^386^ In addition to nanoparticles based on β-gal activity, nanoparticles based on the specific enzyme (MMP-3)^387^ activity of the aging secretion group can also be used to increase the specific delivery of senolytics.

Given the superior properties of natural products,^388^ various natural senolytic agents have been successfully discovered (Fig. 12, Table 2). Among them, fisetin,^30,389^ procyanidin C1,^390,391^ flavonoid 4,4’-dimethoxychalcone,^392^ and cardiac glycosides^393^ have shown strong senolytic efficacy. Considering the encouraging performance of senolytics, more superior senolytics are expected to be identified.

#### Senolytic CAR-T

Senolytic chimeric antigen receptor (CAR)-T (CAR-T) cell therapy is an emerging antiaging strategy that genetically engineers T cells to target senescent cells to delay or reverse aging-related diseases. Studies have revealed that there is a protein marker of urokinase plasminogen activator receptor (uPAR) on the surface of senescent cells and that uPAR-positive senescent cells accumulate during aging.^36^ On the basis of this marker, a chimeric peptide, E16-uPA24, that specifically targets senescent cells was constructed.^394^ This small molecule can promote immune cells to recognize and kill senescent cells, thereby effectively eliminating senescent cells. Based on this, an approach based on CAR-T cells has been developed to eliminate senescent cells. uPAR-28z CAR-T cells were generated, and a single injection effectively eliminated senescent cells and improved nonalcoholic fatty liver disease (NASH)-induced liver fibrosis in young mice.^37^ Further studies have shown that senolytic uPAR-CAR-T-cell therapy can safely and effectively eliminate senescent cells and prolong the lifespan of aged mice.^395^ In addition, senolytic uPAR-CAR-T-cells have been shown to promote intestinal stem cell regeneration and restore the intestinal niche.^396^ Notably, natural killer group 2 member D ligands (NKG2DLs) are upregulated in various tissues of aging mice and aging nonhuman primates.^397^ Accordingly, NKG2D-CAR-T-cell therapy has been developed to selectively target and eliminate senescent NKG2DL-expressing cells in mice and nonhuman primates for the treatment of aging and age-related diseases driven by aging.^397,398^ At present, only two validated senescence markers are used for CAR-T-cell therapy, and more specific targets in aging tissues need to be identified in the future. Senolytic CAR-T-cell therapy represents a groundbreaking advancement in geroscience, offering targeted clearance of senescent cells through precision immunotherapy. While challenges persist in cost optimization, safety profiling, and biomarker heterogeneity, its unique advantages of sustained efficacy and systemic action position this modality as a promising candidate for addressing age-related pathologies. Future directions should focus on integrating multiomics platforms (e.g., single-cell transcriptomics and spatial proteomics) to refine target selection paradigms while leveraging engineering innovations to lower translational barriers, ultimately bridging the gap between preclinical validation and clinical implementation.

Owing to the different mechanisms of action of senolytic drugs, the clearance of senescent cells also varies. Additionally, there are multiple problems that need to be solved. First, the ability of senolytics to selectively eliminate senescent cells are not high. Currently, the majority of senolytics still cause damage to normal cells. More importantly, senolytic drugs are unable to distinguish between beneficial aging cells and harmful aging cells in tissue repair. Second, the optimal therapeutic time is uncertain. Senolytic drugs can deplete stem cells after premature therapy, whereas late use can reduce efficacy. Third, the residual decomposition products cannot be removed in time after the senescent cells are eliminated, and the resulting tissue voids cannot be repaired. Finally, the distribution of senescent cells varies in different tissues and organs. How to improve the ability of senolytic drugs to target and penetrate organs remains to be determined.

### Senomorphics

Although senolytic agents have shown superior effects in eliminating senescent cells and delaying aging, their low specificity and side effects still need to be addressed. Senomorphic therapy delays the aging process by inhibiting the SASP, offering promises in scenarios where the transient beneficial functions of senescent cells are preserved. This approach complements senolytic therapy, which directly eliminates senescent cells, creating a synergistic framework for aging intervention. The core function of senomorphic agents is to regulate the SASP rather than directly eliminating senescent cells. These agents mainly include those that inhibit SASP-related signaling pathways, regulate epigenetic modifications, enhance autophagy, improve mitochondrial function, etc. Furthermore, target individual cytokines (anti-IL-1α glucocorticoids, anti-IL-6 simvastatin/haritaki) and small molecules that modulate upstream regulators such as p53 (nutlin-3a) or inhibit LINE-1-driven inflammatory signaling (nucleoside reverse transcriptase inhibitors like lamivudine) also function as SASP inhibitors.^399^ As the research is at an early stage, there are different thoughts regarding whether certain compounds can be defined as senomorphic agents. Further research is needed to elucidate their mechanism of action before we can be more definitive in defining them. Here, we focused on introducing several highly promising agents.

#### Rapamycin

Rapamycin is also an effective SASP inhibitor that controls the SASP through the regulation of MAPKAPK2 kinase translation by 4EBP1.^400^ Later-life rapamycin feeding increases lifespan by 14% in females and 9% in males,^401^ with a sex difference.^402^ However, long-term administration of rapamycin has significant side effects (including skin and metabolic disorders) and inhibits mTORC2.^140^ Given the side effects of mTORC1 inhibitors such as rapamycin, dietary restriction has shown superior health and longevity extension performance by inhibiting mTORC1. The breakdown metabolism of branched-chain amino acids (BCAAs) is a noteworthy regulator of physiological aging, and blocking the accumulation of BCAAs can alleviate the SASP by inhibiting mTORC1.^403^ A protein-restricted (BCAA leucine, isoleucine, and valine) diet prolongs the healthspan and lifespan of mice by reducing mTORC1 activity in male mice.^404^ In addition, ketogenic diets have also been shown to extend the longevity of mice by suppressing mTORC1 activity.^405,406^ Drug combinations that target nutrient sensing have shown superior performance in terms of lifespan. The combination of trametinib, the mTORC1 inhibitor rapamycin, and lithium prolongs the lifespan of *Drosophila* by 48%.^407^ Therefore, the combination of drugs that act simultaneously on different nutrient-sensing signaling factors and rapamycin can synergistically exert their beneficial effects, providing directions for future research on antiaging combinations.

#### Metformin

Accumulating evidence suggests that metformin can delay aging in nematodes and mice. Mechanistically, metformin prolongs lifespan mainly by activating AMPK to inhibit the SASP. Dysfunction of AMPK in nematodes can eliminate the longevity-prolonging function caused by metformin. Presenilin enhancer 2 (PEN2) is regarded as the target of metformin.^408^ Metformin binds to PEN2 to activate AMPK and cross-links to the lysosomal glucose-sensing pathway through ATP6AP1, thereby delaying aging.^408^ Metformin can reduce the SASP by enhancing autophagy and mitophagy and improve the aging of VSMCs and auditory cells.^409^ In addition, metformin can enhance the anticancer efficacy of CDK4/6 inhibitors by inhibiting SASP.^410^

#### Lithocholic acid

Lithocholic acid (LCA) is a secondary bile acid that is synthesized mainly in the liver and is produced in the gut through bacterial metabolism. It plays a variety of roles in bile, such as promoting fat digestion and absorption, regulating blood lipids, promoting cell proliferation, inhibiting inflammation and protecting the gastric mucosa. Previous studies have shown that LCA can extend the lifespan of yeast and *Drosophila melanogaster*.^411^ Calorie restriction is widely accepted as an approach to prolong life and delay aging. However, long-term dietary restriction is challenging to implement broadly in the elderly population, as it may lead to malnutrition and muscle wasting. Thus, identifying interventions that can extend healthspan and lifespan without requiring caloric restriction has become a key research goal. LCA acts as a caloric restriction mimetic to prolong the lifespan of *C. elegans*, *Drosophila*, and mice.^25^ Mechanistically, LCA targets the protein TULP3 to activate the sirtuin-v-ATPase-AMPK signaling axis, thereby exerting antiaging effects through conserved metabolic regulatory pathways.^26^ More importantly, unlike caloric restriction, LCA does not induce the adverse effect of muscle loss observed in caloric restriction, offering a superior strategy to enhance healthspan. Notably, long-term administration of high-dose LCA exerts adverse effects on the liver, disrupting phospholipid-sphingolipid homeostasis and thereby inducing cholestasis, which severely impairs hepatic function.^412^ Therefore, more rigorous clinical trials are needed before approval can be obtained.

### Senoreverse

In 2006, Satoshi Yamanaka announced at the International Society for Stem Cell Research that a group of genetically engineered cells could be induced into iPSCs. By importing four transcription factors, OSKM, senescent cells are successfully restored to the ESC state.^413^ The periodic short-term expression of OSKM for partial reprogramming improves the physiological properties of cellular senescence and extends the lifespan of mice by 30%.^17,18^ The introduction of Oct4, Sox2, and Klf4 (OSK) restores the DNA methylation pattern and transcriptomes of young mice, promotes axonal regeneration after injury, and reverses visual loss in glaucoma mice and aged mice.^20^ Additionally, the expression of Oct4, Sox2, and Klf4 can also reverse the changes in epigenetic information caused by aging,^62^ prolonging the median remaining longevity of aged mice. To improve the precision of OSK partial reprogramming and reduce potential tumor risk. Using an adeno-associated virus as a vector, OSK was precisely delivered to senescent cells with a highly active *Cdkn2a* promoter, an important marker of cellular senescence.^19^ This “precise targeting” mechanism ensures that OSK functions only in senescent cells that highly express *Cdkn2a*, restoring these senescent cells to a more youthful state and prolonging the lifespan of aging mice.^19^ However, this intervention faces challenges of safety and limited efficacy for inducing rejuvenation in vivo. Therefore, the identification of novel senescence reversal factors is needed. High-throughput screening identified a small TERT-activating compound that could increase TERT transcription in senescent cells, restore it to the physiological expression level in young cells, promote telomere synthesis, reduce DNA damage signals at telomeres, and thus reverse cellular senescence.^47^ Reversine, a novel inhibitor of aurora kinase, can restore the impaired myogenic differentiation potential of senescent myoblasts and dysfunctional mitochondria through the reactivation of autophagy.^414^ These findings suggest that cellular senescence can be reversed by single small-molecule treatment. Regular intravenous injection of sEVs from young mouse plasma into old mice significantly prolonged the median lifespan and improved survival. Young sEVs stimulate PGC-1α expression through their miRNA payload, which helps to improve mitochondrial function and alleviate mitochondrial defects in aging tissues.^203^ In addition, young sEVs significantly reduced the vulnerability index of aged mice, suggesting a substantial reversal of biological age. In 2025, Guangju Ji’s team creatively proposed the “senoreverse” strategy to reverse aging.^38^ It has the potential to revert senescent cells to an earlier state via continuous delivery of miR-302b derived from embryonic stem cell exosomes.^38^ miR-302b regulates the cell cycle by targeting *Cdkn1a* and *Ccng2*, restores the proliferative capacity of senescent cells, significantly extends lifespan and improves physiological function in aging mice.^38^ Unlike traditional approaches such as senolytics or senomorphics, the opposite focuses on restoring the function of senescent cells and mitigates the risks of tissue damage and immune suppression.

Interestingly, in addition to genetic or pharmacological means, mechanical stress has shown promising potential in reversing aging. Triboelectric stimulation rejuvenates senescent BMSCs and restores their osteogenic and chondrogenic potential by increasing MDM2-dependent p53 degradation.^415^ In recent years, low-intensity pulsed ultrasound has shown superior therapeutic potential in a variety of aging-related diseases. Low-frequency sonication allowed the expansion of primary cells and stem cells beyond the normal replication limit without affecting the phenotype.^416^ Low-intensity pulsed ultrasound enhances the migration of immune cells and the phagocytosis of M1 macrophages on senescent cells by selectively destroying the cell membrane structure and SASP secretion of senescent cells.^417^ In addition, low-frequency ultrasound treatment of nongrowing senescent cells restored the growth of senescent cells by increasing autophagy and Sirtuin 1 expression.^416^ However, there are still many problems to be solved in reversing aging by mechanical stress. First, the response threshold of different tissues to mechanical stress varies greatly and needs to be precisely regulated to avoid excessive stress leading to damage. Second, how mechanical signals cross-regulate with metabolic and epigenetic networks still needs to be further studied. Finally, there is a need to develop noninvasive and controllable mechanical intervention devices.

### Dietary Interventions and natural supplements

While great deal of research efforts were invested in therapeutic agents to modulate aging processes. To achieve longevity and healthy aging, foods and healthy life style play fundamental roles. The macronutrients and micronutrients in our diet not only ensure sufficient nutritional needs but also have Recent studies have shown that dietary fiber may delay aging.^418^ While healthy foods containing phytochemicals that may suppress systemic inflammation is beneficial to healthy aging, unhealthy foods may promote inflammation and speeding aging and its related diseases.

Different dietary patterns, such as Mediterranean and ketogenic diets, can activate various mechanisms that increase health benefits from nutrition and reduce the likelihood of age-related diseases. A 30-year study involving more than 100,000 participants revealed that, overall, a diet rich in fruits, vegetables, nuts, and unsaturated fats was most closely associated with healthy aging. In contrast, a higher intake of trans fats was strongly correlated with a reduced likelihood of healthy aging.^419^ In addition to these dietary patterns, structured approaches such as caloric restriction, intermittent fasting (IF), and time-restricted feeding are receiving attention for their ability to influence biological aging. Caloric restriction signifies the consumption of 20–40% fewer calories than the energy required without sacrificing nutrition.^420^ In 1935, McCay and colleagues first identified the connection between caloric restriction and extended lifespan. They reported that albino rats with reduced caloric intake enjoyed significantly longer, healthier lives and slower growth than those given ad libitum access to food.^421^ Research conducted across various species, including mice,^422^ nematodes, *Drosophila* flies, and rhesus monkeys,^423^ has consistently supported McCay’s observations. Despite promising results in preclinical models and short-term human trials, several challenges must be addressed regarding these dietary interventions. A significant challenge is finding the optimal caloric intake for individuals to avoid underfeeding or overfeeding. Personalization is crucial, yet factoring highly complex factors such as genetics, sex, metabolic rate, microbiome composition, socioeconomic status, circadian rhythms, and cultural influences remains challenging. These variables significantly affect individual responses, limiting the applicability of generalized dietary recommendations. Another challenge is conducting studies on healthspan or lifespan extension that are translatable to humans, given that humans have a significantly longer lifespan than the experimental models used. Furthermore, more stringent experimental methods, such as clearly defining caloric intake among experimental groups and controls, should be implemented. In summary, this highlights the need for further research focusing on genetic and environmental diversity, as well as stricter enforcement of caloric intake guidelines that can distinguish between actual caloric restriction and other dietary variations. Apart from multifactorial interventions, alternatives to caloric restriction can include environmental conditioning and caloric restriction mimetics, including rapamycin, metformin, or a blend of micronutrients and bioactives, including quercetin, rutin, curcumin, genistein, resveratrol, epigallocatechin gallate, gallic acid, betaine, theanine, γ-aminobutyric acid, diallyl sulforated compounds, NAD^+^ precursors, coenzyme Q10, vitamins, plant polysaccharide, plant-derived peptides, and others.^424^ Supplementation of free amino acids, such as glycine, proline and arginine, can also significantly improve the aging state. Here we have described some important natural dietary supplements, including Urolithin A, Spermidine, and Ergothioneine.

#### Urolithin A

Urolithin A is generated by the metabolism of ellagitannins and ellagic acid via gut microbes and is found mainly in pomegranates, nuts, and some berries. Urolithin A can inhibit the SASP through a variety of pathways, thereby improving aging-related diseases. In the *C. elegans* model, urolithin A activates the AMPK signaling pathway and significantly promotes mitophagy, which prolongs lifespan by 45.4%.^425^ Similarly, muscle endurance is increased by 40% in aged mice after urolithin A treatment.^425^ Defects in mitophagy play important roles in the progression of Duchenne muscular dystrophy (DMD). Therapy with urolithin A restores mitophagy in DMD worms and mice, as well as in primary myoblasts from DMD patients, thereby restoring muscle function and improving survival in the DMD mouse model.^426^ In an aged mouse model, urolithin A-induced mitophagy weakened cGAS/STING activation and improved neurological deterioration.^427^ In addition, treatment with urolithin A can improve the mitochondrial function of aging HSCs through promoting mitophagy, thereby restoring these cells to their youthful hematopoietic function levels.^428^

#### Spermidine

Spermidine, a naturally occurring polyamine, is widely distributed in plant and animal tissues (e.g., cheese, legumes, mushrooms) and human cells, and its levels are maintained through dietary intake or endogenous synthesis. Spermidine levels decline progressively with age and are closely associated with the development of age-related diseases. Spermidine delays aging through multiple pathways, such as the activation of autophagy, the regulation of epigenetic modifications, the improvement of mitochondrial function and the inhibition of inflammation, and has shown potential for improving healthspan in model organisms (yeasts, *Drosophila*, nematodes, and mice)^429^ and humans. By activating mitophagy and enhancing mitochondrial respiration, spermidine mitigates brain aging and improves cognitive function. Mechanistically, dietary spermidine crosses the blood-brain barrier in mice, increasing hypusination of eukaryotic translation initiation factor 5A (EIF5A) and mitochondrial function in the hippocampus while reducing mitochondrial decay.^430,431^ This effect requires the autophagy regulator ATG7 as well as the mitophagy mediators PARKIN and PINK1. Inhibition of the enzymes required for EIF5A hypusination abolishes fasting-mediated autophagy enhancement and longevity extension.^430,431^ Furthermore, spermidine delays oocyte aging by activating mitophagy and maintaining mitochondrial function, improves the quality of aged oocytes after ovulation, and promotes fertility in aged female mice,^432,433^ which can potentially be applied to human-assisted reproductive technology.^433^ Overall, spermidine, with its natural origin, few side effects, and convenient access through diet or supplements, is poised to become a pivotal tool for future pro-longevity interventions.

#### Ergothioneine

Ergothioneine (EGT) is a natural sulfur-containing amino acid that is widely present in mushrooms (such as shiitakes and Pleurotus edodes), animal livers, and legumes. As humans cannot synthesize EGT endogenously, it must be obtained through diet or supplementation. Characterized by a unique imidazole ring structure, EGT has potent antioxidant and anti-inflammatory properties and selectively accumulates in highly metabolic tissues such as the brain, kidneys, and retina. Notably, circulating EGT levels decline progressively with age, correlating negatively with the risk of age-related diseases.^434^ In recent years, its antiaging potential has emerged as a hotspot, particularly for extending healthspan, improving mitochondrial function, and regulating oxidative stress pathways. EGT supplementation prolongs the lifespan and promotes healthy aging in *C. elegans* and mice.^435^ Importantly, dietary EGT intake inhibits age-related degeneration in multiple organs, including the kidneys, liver and brain, while ameliorating age-associated impairments in learning and memory capabilities. EGT can reduce the damage to hippocampal neurons induced by D-galactose and improve the learning and memory ability of mice by regulating the AMPK/SIRT1/PGC-1α pathway.^436^ Recent studies have shown that ergothioneine (ET) acts as an alternative substrate for cystathionine γ-lyase, stimulating hydrogen sulfide production.^437^ This process induces persulfidation of the critical cysteine residue C243 in cytosolic glycerol-3-phosphate dehydrogenase (cGPDH), thereby increasing its catalytic activity.^437^ The resulting increase in cGPDH activity promotes NAD⁺ regeneration and maintains cellular energy metabolism.^437^ This explains why EGT mainly enhances exercise endurance, improves metabolic health, and delays the occurrence of aging-related diseases through persulfidation. Dietary supplementation with EGT significantly enhances exercise endurance in mice. Proteome-wide thermal stability analysis revealed that EGT directly binds to and activates the mitochondrial enzyme 3-mercaptopyruvate sulfurtransferase (MPST), thereby increasing mitochondrial respiration and improving exercise performance.^438^ This is the first physiologically relevant EGT target and establishes the EGT-MPST axis as a regulator of mitochondrial function, ameliorating aging-related diseases. However, the long-term efficacy of caloric restriction mimetics as a geroprotector and their safety require further investigation. Ultimately, the goal is to strike a balance between the benefits of longevity and practicality while developing interventions for aging and health that are both accessible and sustainable.

Although these compounds show potential antiaging properties. There are some negative effects of some of the agents. For example, supplementation of β-nicotinamide mononucleotide/nicotinamide riboside (NMN/NR) in aged mice can increase the level of NAD^+^ and extend the lifespan of mice.^439^ However, nicotinamide nucleoside NR supplementation increased the incidence of tumors by 27% and increased the incidence of brain metastases by 227.2% compared with controls.^440^ The carcinogenic risk of antiaging drugs is a core contradiction in the field of antiaging medicine, which essentially stems from the deep interweaving of aging and cancer in biological mechanisms. Both share key pathways such as cell proliferation regulation, DNA damage repair, and immune surveillance. Antiaging drugs often work by promoting cell repair and delaying apoptosis, but excessive inhibition of apoptosis or enhanced proliferation may lead to the survival and accumulation of abnormal cells, such as cancer cells. Some drugs delay aging by activating DNA repair pathways, yet abnormal repair mechanisms may mask gene mutations in cancer cells and accelerate tumor development. The immune system can both eliminate senescent cells and recognize and kill cancer cells. If drugs excessively suppress immune inflammation (such as rapamycin), they may weaken the surveillance against cancer cells, resulting in cancer cell metastasis. In addition, telomerase activators delay aging by extending telomeres, but this mechanism inherently carries carcinogenic risks. Activating telomerase may accelerate the proliferation of existing cancer cells or induce the carcinogenesis of normal cells. Like any other therapeutic agents, both safety and efficacy shall be fully studied and the therapeutic windows to be determined before safe dosages can be determined.

## Artificial intelligence (AI) assists in the development of antiaging agents

AI technology has been applied to the discovery of senolytic agents. It is very effective in helping us discover new candidate drugs, especially in the early stages of drug discovery. Scientists have developed an AI algorithm to screen a collection of more than 4300 compounds and have successfully identified 21 potential antiaging candidate compounds. These candidate drugs have the ability to eliminate dysfunctional cells without damaging healthy cells. Further experiments revealed that the natural products periplocin, oleandrin and ginkgetin can be used as senolytic agents.^441^ Another study using deep neural networks revealed that the small molecules RDK20733377, BRD-K56819078 and BRD-K44839765 can selectively induce the apoptosis of senescent cells.^442^ A successful morphology-based convolutional neural network system (deep learning-based senescence scoring system by morphology, Deep-SeSMo) was developed to evaluate the effects of antiaging agents.^443^ The natural products terreic acid and daidzein were successfully screened as antiaging agents through the Deep-SeSMo system. Transcriptome sequencing experiments confirmed that drugs reduce the senescent phenotype of cells by restraining inflammation.^443^ AgeXtend, an AI-powered multimodal platform for geroprotective factor discovery,^444^ integrates multidimensional biological activity profiles of known longevity-related molecules to (1) predict the pharmacodynamic potential of candidate geroprotectors, (2) systematically evaluate pharmacokinetic properties and toxicity profiles, and (3) elucidate molecular targets and mechanisms of action through deep learning-driven pattern recognition. Approximately 1.1 billion compounds were screened via this platform, and multiple candidate compounds with geroprotective properties that demonstrate structural novelty and target specificity were successfully identified.^444^ This computational framework establishes a robust pipeline for accelerating the discovery of novel therapeutic interventions in age-related pathology, providing critical insights for translational gerontology research. In summary, AI has shown outstanding advantages in the discovery of potential novel antiaging agents.

AI has also demonstrated superior performance in discovering aging-related targets. A deep learning-based inflammatory aging clock (iAge) was developed to assess immunosenescence, frailty, multimorbidity, and cardiovascular aging.^445^ The theory behind this “clock” of aging is that the blood levels of particular immune cells and proteins change with age. Through iAge, the interferon-related chemokine CXCL9 is closely related to endothelial cell senescence and promotes the upregulation of multiple inflammatory pathway genes. Reducing CXCL9 enables senescent endothelial cells to restore function.^445^ Notably, other proteomic aging clocks also reveal that CXCL9 is a target of aging.^446^ PandaOmics, an AI-based biological target discovery platform, is available for target identification of age-related diseases. The discovery of KDM1A via PandaOmics has the potential to become a dual target for antiaging and antitumor therapy, with the same therapeutic direction.^447^ Regenerative Bio pioneered an organ aging clock model based on trace cell-free DNA detection and tissue signal decomposition algorithm technology, capturing and analyzing organ aging signals utilizing DNA methylation as a subjective indicator. To better predict the risks of age-related diseases and death, an AI algorithm based on plasma proteomics was established to measure human organ aging, and the aging of 11 major organs was analyzed via this machine learning model.^448^ This machine learning method contributes to the assessment of the degree of organ aging.

In conclusion, AI has demonstrated significant potential in advancing aging research through four primary applications: (1) precise identification of senescent cell populations; (2) computational discovery of novel senolytic compounds; (3) predictive modeling of drug delivery efficiency with concomitant assessment of off-target effects; and (4) integration of multidimensional data such as genomics, epigenetics, metabolomics, and the microbiome to predict individual disease risk, aging speed, and even longevity potential and draw a comprehensive health map for patients. Unfortunately, the employment of AI technology in the pharmaceutical industry continues to face significant barriers, such as low algorithm efficiency, data fragmentation, and insufficient processing capacity, which hinder the advancement of AI technology in the life sciences. Even so, with the continuous advancement of technology and the increase in data resources, the application prospects of AI in drug research and development are still very broad.

## Clinical research and translational applications

### Current trends in clinical trials for aging interventions

#### Senolytic agents in the clinical stage

Several promising strategies against aging have entered clinical trials with preliminary evidence of safety and efficacy in humans (Table 3). A clinical study (NCT02848131) demonstrated that senolytic (D + Q) agents successfully clear senescent cells in diabetic nephropathy patients and significantly relieve the burden of senescent cells in adipose and skin tissues.^449^ Another clinical study (NCT02874989) revealed that D + Q improved physical dysfunction in IPF^31^ patients and was well tolerated.^450^ A phase 1 trial (NCT04063124) of AD revealed that senolytics (D + Q) are safe, feasible and well tolerated.^33,34^ A phase 2 clinical study (NCT04313634) using senolytics (D + Q) to treat bone loss in postmenopausal women showed that patients with higher senescent cell burden had a more significant response to D + Q, manifested as a 34% increase in bone formation and an 11% decrease in bone resorption.^451^ Fisetin, another natural senolytic drug, has also been studied in the clinical stage (Table 3) for the treatment of age-related diseases, and its use is expected to lead to breakthroughs in the clinical research of senolytics. UBX0101, a p53/MDM2 interaction inhibitor, improves osteoarthritis by eliminating senescent synovial cells in the knee joint.^169,452^ Two phase 1 trials from 2018 to 2020 (NCT04229225 and NCT03513016) revealed that UBX0101 was well tolerated.^453^ However, a subsequent phase 2 clinical trial (NCT04129944) failed to demonstrate the efficacy of single-dose, intra-articular dosing of UBX0101 in patients with painful knee osteoarthritis.^454^ This might be caused by factors such as the method of administration, the expectations of patients or researchers, and gender differences in pain reports. UBX1325 (foselutoclax), a novel antiaging small-molecule BCL-xL inhibitor, was the first senolytic agent evaluated for the treatment of DME. The phase I clinical data (NCT04537884) revealed good safety and tolerability, with beneficial and long-lasting effects on visual acuity and macular thickness,^35^ and were shown to restore outer retinal function.^455^ A subsequent phase 2 (NCT04857996) study evaluated the activity of a single intravitreal injection of UBX1325 for the treatment of DME. After 48 weeks of UBX1325 treatment, the best-corrected visual acuity significantly improved, and the central subfield thickness steadily decreased.^456^ Although senolytics have shown preliminary efficacy in clinical studies, long-term evaluation is still lacking. In conclusion, elimination of senescent cells plays a significant role in the treatment of a variety of aging-related pathological states. However, further studies are needed to verify that the underlying senescent cell burden may determine the clinical response to aging.

**Table 3:** Ongoing or completed clinical trials targeting aging and aging-related diseases

Drug types	Intervention	Age-related diseases	Phase	Status	ClinicalTrials ID.
Senolytics	UBX1325 (foselutoclax)	DME	Phase 2	Completed	NCT04857996
		DME or neovascular age-related macular degeneration.	Phase 1	Completed	NCT04537884
		Neovascular age-related macular degeneration	Phase 2	Completed	NCT05275205
		DME	Phase 2	Active, not recruiting	NCT06011798
	UBX0101	Osteoarthritis of the knee	Phase 1	Completed	NCT04229225
		Osteoarthritis of the knee	Phase 1	Completed	NCT03513016
		Osteoarthritis of the knee	Phase 2	Completed	NCT04129944
	Quercetin and Dasatinib	Idiopathic fibrosis	Phase 1	Completed	NCT02874989
		Mild Alzheimer’s disease	Phase 1/2	Enrolling by invitation	NCT04785300
		Nonalcoholic fatty liver	Phase 1/2	Recruiting	NCT05506488
		Alzheimer’s disease	Phase 1/2	Completed	NCT05422885
		Alzheimer’s disease	Phase 1/2	Completed	NCT04063124
		Alzheimer’s disease	Phase 2	Active, not recruiting	NCT04685590
		Chronic kidney disease	Phase 2	Enrolling by invitation	NCT02848131
		Mental disorders	Phase 2	Recruiting	NCT05838560
		Epigenetic aging	Phase 2	Unknown status	NCT04946383
		Osteoporosis	Phase 2	Recruiting	NCT06018467
		Improve frailty	Phase 2	Recruiting	NCT04733534
		Chemo resistance in triple negative breast cancer	Phase 2	Recruiting	NCT06355037
		Improve skeletal health in older	Phase 2	Completed	NCT04313634
	Fisetin	Carpal tunnel syndrome	Phase 2	Active, not recruiting	NCT05416515
		Peripheral artery disease	Phase 2	Recruiting	NCT06399809
		Improve vascular function	Phase 1/2	Recruiting	NCT06133634
		Osteoarthritis	Phase 1/2	Active, not recruiting	NCT04815902
		Common variable immunodeficiency	Phase 2	Enrolling by invitation	NCT05593588
		Sepsis	Phase 2	Recruiting	NCT05758246
		Osteoarthritis-related articular cartilage degeneration	Phase 1/2	Completed	NCT04210986
		Meniscus repair	Phase 2/3	Not yet recruiting	NCT05505747
		Frailty and markers of inflammation, insulin resistance, and bone resorption	Phase 2	Enrolling by invitation	NCT03675724
		Insulin resistance inflammation, bone resorption and Physical dysfunction	Phase 2	Enrolling by invitation	NCT03430037
		Improve frailty	Phase 2	Recruiting	NCT04733534
Senomorphics	Procyanidin C1+Cellumiva (grape seed extract, Pterostilbene and Spermidine)	Skin rejuvenation	Not Applicable	Completed	NCT06641869
	Resveratrol	Improved performance in the elderly	Phase 1	Completed	NCT01126229
		Enhance vitality and vigor in elders	Phase 2	Completed	NCT02123121
	Urolithin A	Evaluate the safety, tolerability, pharmacokinetics and pharmacodynamic profile in healthy elderly	Phase 1	Completed	NCT02655393
		Improve muscle performance	Phase 2	Completed	NCT03464500
		Counteract age-associated muscle decline	Phase 2	Completed	NCT03283462
		Antiaging efficacy of one face care cosmetic product	Not Applicable	Recruiting	NCT06619457
		Mitochondrial quality in muscle of frail older adults	Not Applicable	Recruiting	NCT06556706
		Glucose metabolism in healthy adults 55 >= years old	Not Applicable	Recruiting	NCT06274749
		Middle-aged adults with obesity	Not Applicable	Recruiting	NCT05921266
		Boost immune health	Not Applicable	Completed	NCT05735886
		Skeletal muscle function, iron metabolism and endurance performance	Not Applicable	Completed	NCT04783207
	Metformin	Aging people with multiple sclerosis	Phase 2	Not yet recruiting	NCT06463743
		Multiple sclerosis brain remyelination and neurodegeneration	Phase 2	Recruiting	NCT05893225
		Progressive multiple sclerosis	Early Phase 1	Recruiting	NCT05349474
		Evaluating the geroprotective effect	Phase 2	Not yet recruiting	NCT06459310
		aging study	Phase 3	Unknown status	NCT04264897
		Antiaging, pro-autophagy effects in adults with prediabetes	Phase 3	Completed	NCT03309007
		Muscle health of older adults	Early Phase 1	Active, not recruiting	NCT03107884
		Longevity study	Phase 4	Completed	NCT02432287
		Alzheimer’s dementia	Phase 2/3	Active, not recruiting	NCT04098666
		Amnestic mild cognitive impairment	Phase 2	Completed	NCT00620191
		Parkinson’s disease	Phase 2	Recruiting	NCT05781711
	Everolimus	Aging study	Phase 2	Recruiting	NCT05835999
		Bone loss in postmenopausal women	Phase 2	Recruiting	NCT06789900
	Rapamycin	Pharmacokinetics and pharmacodynamics	Phase 1/2	Not yet recruiting	NCT06727305
		Reducing clinical measures of aging in an older adult population	Phase 2	Completed	NCT04488601
		Ovarian aging	Phase 2	Active, not recruiting	NCT05836025
		Alzheimer’s and cognitive health	Phase 2	Recruiting	NCT04629495
		Alzheimer’s disease	Phase 1/2	Enrolling by invitation	NCT06022068
		Aging of the skin	Phase 1/2	Completed	NCT03103893
	Vitamin D and omega-3	Delay biological aging by three to four months; Reduce the risk of cancer in people over 70 years of age by 61%	Phase 3	Completed	NCT01745263
		Aging and longevity	Observational	Unknown status	NCT05062018
	Omega 3	Aging-related cognitive	Not Applicable	Completed	NCT04484454
		Aging-related cognitive	Phase 1	Unknown status	NCT05041088
		Healthy aging	Not Applicable	Recruiting	NCT06150261
		Aging brain	Phase 3	Unknown status	NCT00996229
	Acarbose	Longevity	Phase 2	Completed	NCT02953093
		Antiaging effects	Phase 2	Completed	NCT02865499
	Pyrroloquinoline quineone, PQQ	Mild cognitive impairment in elderly	Not Applicable	Completed	NCT05910047
		Healthy postmenopausal women	Not Applicable	Recruiting	NCT06748989
	Ergothioneine	Skin condition	Not Applicable	Completed	NCT06886061
		Kidney failure	Not Applicable	Not yet recruiting	NCT06487546
		Delay cognitive decline	Phase 2	Completed	NCT03641404
Endogenous metabolites	Alpha-ketoglutarate	Inflammatory syndrome in surgery-colorectal	Phase 3	Recruiting	NCT06646809
		BiologicaL agE in middle-aged adults	Phase 2	Recruiting	NCT05706389
	Spermidine	Bioavailability of spermidine in healthy males	Not Applicable	Active, not recruiting	NCT06017219
		Metabolic responses	Not Applicable	Active, not recruiting	NCT05459961
		Improving vaccination in older adults	Not Applicable	Active, not recruiting	NCT05421546
		Elderly patients with coronary artery disease	Phase 2	Recruiting	NCT06186102
		Anti-hypertension	Phase 3	Unknown status	NCT04405388
		Lower all-cause mortality	Not Applicable	Completed	NCT03378843
		Verbal memory and inflammation	Phase 2	Completed	NCT03094546
		Memory performance in older adults at risk for dementia	Phase 2	Completed	NCT02755246
	Nicotinamide riboside, NR	Immune thrombocytopenia	Phase 1/2	Not yet recruiting	NCT06776510
		Atypical parkinsonism	Phase 2	Recruiting	NCT06162013
		Brain vascular health in aging	Phase 4	Recruiting	NCT05483465
		Prevent progressive neurological disease	Phase 2	Active, not recruiting	NCT04870866
		Huntington’s disease	Phase 2	Not yet recruiting	NCT06853743
		Peripheral artery disease	Phase 1/2	Active, not recruiting	NCT06534944
		Promote healthy longevity	Not Applicable	Recruiting	NCT06425042
		Therapy against functional decline in aging	Phase 2	Recruiting	NCT06208527
		Alzheimer’s disease	Phase 2	Recruiting	NCT05617508
		Elevated systolic blood pressure and arterial stiffness in middle-aged and older adults	Phase 2	Recruiting	NCT03821623
		Bone, skeletal muscle and metabolic functions in aging	Not Applicable	Completed	NCT03818802
		Drug naïve Parkinson’s disease	Not Applicable	Completed	NCT03816020
		Li-Fraumeni syndrome	Phase 1/2	Completed	NCT03789175
		Early Parkinson’s disease	Phase 3	Recruiting	NCT03568968
		Improving memory and brain blood flow in older adults with mild cognitive impairment	Phase 1/2	Completed	NCT03482167
		Skeletal muscle metabolic, increases NAD^+^ metabolism in muscle and decreases circulating inflammatory cytokine	Phase 2	Completed	NCT02950441
		Systolic heart failure	Phase 1/2	Completed	NCT03423342
		No changes in insulin sensitivity, glucose metabolism, or mitochondrial respiration	Not Applicable	Completed	NCT02303483
		Increased NAD^+^in whole blood	Phase 2	Completed	NCT02712593
		Macula off retinal detachment	Phase 4	Not yet recruiting	NCT06587945
		Reduce epigenetic aging in chronic obstructive pulmonary disease	Not Applicable	Completed	NCT04990869
		Improved 6-min walk in PAD	Phase 3	Completed	NCT03743636
		Augmente the blood NAD^+^ metabolome	Not Applicable	Completed	NCT05344404
		Increase blood NAD^+^ concentrations in older adults	Not Applicable	Completed	NCT02942888
		Augment neuronal NAD^+^ levels	Phase 1/2	Completed	NCT02921659
		NR and pterostilbene, decrease alanine aminotransferase and gamma-glutamyltransferase	Not Applicable	Completed	NCT03513523
		NR and pterostilbene, increases whole blood NAD^+^ levels	Not Applicable	Completed	NCT03176628
		No improvement in MuSC pool recruitment	Not Applicable	Completed	NCT03754842
		Increase skeletal muscle NAD^+^ metabolites	Not Applicable	Completed	NCT02835664
		Improve mitochondrial respiration and attenuate proinflammatory activation	Early Phase 1	Completed	NCT03727646
		Huntington’s disease	Phase 2	Recruiting	NCT06853743
		Idiopathic pulmonary fibrosis	Phase 2	Not yet recruiting	NCT06567717
		Progressive multiple sclerosis	Phase 2	Recruiting	NCT05740722
		Ulcerative colitis	Not Applicable	Recruiting	NCT05561738
		Cognition and sleep	Not Applicable	Recruiting	NCT05500170
	MIB-626	Friedreich’s ataxia	Phase 2a	Completed	NCT04817111
		Alzheimer’s disease	Phase 1/2	Recruiting	NCT05040321
		Diabetes kidney disease	Phase 2a	Recruiting	NCT05759468
	Nicotinamide Mononucleotide, NMN	Increased blood NAD levels, maintained walking speed, and improved sleep quality in older adults	Not Applicable	Completed	UMIN000047871
		Alleviating arterial stiffness	Not Applicable	Completed	UMIN000045205
		Safety evaluation	Not Applicable	Completed	UMIN000043084
		Sleep quality, fatigue, and physical performance in older Japanese adults	Not Applicable	Completed	UMIN000038097
		Enhanced O2 utilization of the skeletal muscle	Not Applicable	Completed	ChiCTR2000035138
		Increases muscle insulin sensitivity in prediabetic women	Not Applicable	Completed	NCT03151239
		Antiaging supplement	Phase 2	Completed	NCT04823260
		Efficacy and safety	Not Applicable	Completed	NCT04228640
		Aging related research	Not Applicable	Active, not recruiting	NCT06592859
		Metabolic flux	Phase 1	Not yet recruiting	NCT06882096
		Mild ulcerative colitis	Not Applicable	Recruiting	NCT06214078
		Promote the rejuvenation of the peripheral blood immune cells	Early Phase 1	Not yet recruiting	NCT05984550
		Polycystic ovary syndrome	Not Applicable	Recruiting	NCT05305677
		Hypertensive	Phase 4	Unknown status	NCT04903210
	Coenzyme Q10 (CoQ10)	Aging study	Not Applicable	Completed	NCT02012322
		Healthy older adults	Not Applicable	Unknown status	NCT05500742

#### Senomorphic agents in the clinical stage

Unlike senolytics, senomorphics offer a complementary strategy to preserve tissue homeostasis while reducing chronic inflammation. Here, we highlight emerging senomorphics in clinical stages and their translational potential. A phase 1 study (NCT02655393) with single or multiple doses over 4 weeks demonstrated favorable safety profiles and improved molecular signatures of mitochondrial and cellular health in healthy sedentary older adults.^457^ Two independent phase 2 clinical studies (NCT03464500, NCT03283462) of urolithin A supplementation revealed that 4 months of urolithin administration enhanced muscle endurance and offset age-related muscle decline.^458,459^ Metformin is the drug of choice for the treatment of type 2 diabetes and has shown definite efficacy in delaying aging in preclinical studies. Mendelian randomization analysis (UK Biobank, *n* = 321,412) revealed that metformin promoted healthy aging through its targets glycerol-3-phosphate dehydrogenase 1 (GPD1) and AMPKγ2.^460^ A phase 1 trial (NCT03107884) revealed that metformin treatment reduced primary muscle fibroadipogenic senescent marker levels of progenitors and decreased type I muscle fiber atrophy.^461^ However, metformin may interfere with the improvement of some physiological functional parameters, such as whole-body insulin sensitivity and cardiorespiratory fitness, and may have a negative effect on the hypertrophic response in healthy elderly individuals.^462^ In addition, owing to the heterogeneity of aging, metformin treatment does not improve physical function in the elderly population with sarcopenia and frailty and is poorly tolerated in this population (ISRCTN29932357).^463^ Hence, further studies are needed before metformin can be widely used as a treatment to delay aging. Clinical studies have shown that mTOR inhibition plays an important role in delaying aging. The phase 2b and phase 3 studies of the mTOR inhibitor RTB101 showed that RTB101 could improve immune function in older adults (NCT03373903, ACTRN12619000628145). In a phase 2a study (ACTRN12613001351707), the TORC1 inhibitors BEZ235 and RAD001 showed potential to improve immune function and reduce infection rates in older adults.^464^ In addition, topical rapamycin treatment reduces the levels of aging markers in aged skin tissue and improves skin health (NCT03103893).^465^ Although clinical studies of senomorphics have revealed phenotypes associated with delayed aging, there are still some cases with insignificant effects and even negative health effects. Therefore, more rigorous and long-term clinical trials are needed for further verification.

#### Endogenous metabolites in the clinical stage

Endogenous metabolites have shown promising longevity potential in preclinical studies. At present, a variety of endogenous metabolites, including α-KG, spermidine and NAD^+^ precursors, have entered the clinical stage for the treatment of aging-related diseases (Table 3). Safety evaluation of a 28-day high-dose (40 mg/d) spermidine supplement in healthy elderly men revealed good safety and tolerability (NCT05459961).^466^ There is a robust association between a spermidine-rich nutritional diet and improved survival in humans (NCT03378843).^467^ However, a 12-month phase 2b (NCT094546) study of dietary spermidine supplementation revealed no changes in memory or biomarkers in older adults, possibly because of the lower spermidine dose (0.9 mg/day)30.^468^ As an important precursor of NAD^+^, long-term NR supplementation was well tolerated, effectively promoted NAD^+^ metabolism and reduced inflammatory levels (NCT02921659, NCT03727646).^439^ In addition, NR supplementation can also increase NAD^+^ levels in the brain and reduce the levels of inflammatory cytokines in the cerebrospinal fluid, and NR can be used as a potential neuroprotective agent for PD (NCT03816020, NCT05344404).^469,470^ A 6-week treatment with NR (NCT04990869) increased NAD^+^ levels in whole blood by more than twofold and reduced epigenetic aging in patients with COPD.^471^ In elderly patients with mild cognitive impairment, 10 weeks of NR supplementation significantly increased blood NAD^+^ concentrations. However, these clinical trials have a short period and a small number of participants, and more in-depth clinical studies are needed before approval.

Owing to the heterogeneity and individual differences in aging, current clinical drugs cannot precisely target senescent cells and have frequent off-target effects, resulting in poor clinical efficacy. In addition, the clinical dosage, individual variation, insufficient sample size, improper selection of endpoint indicators and short follow-up time may all be potential factors for the failure of antiaging clinical trials. Therefore, critically analyzing negative clinical results is necessary to avoid misleading conclusions, standardize the clinical relevance of endpoint indicators, and promote the rational development of the antiaging field.

In conclusion, it is necessary to explore the synergistic effects of existing natural compounds, reduce the limitations of single therapies, optimize dosing regimens, and carry out large-scale long-term studies. In the future, it is necessary to integrate multiomics data, develop precision delivery technologies, and promote the translation of basic research to clinical application through interdisciplinary cooperation to provide innovative solutions for healthy aging.

### Challenges in translating antiaging therapies to humans

The translation of healthy longevity and antiaging therapies from laboratory research to clinical applications faces multifaceted challenges. First, ensuring clinical safety and efficacy remains a major hurdle. Current technologies, including senolytic therapies, senomorphic interventions, and stem cell therapies, carry inherent risks. Senolytic agents exhibit limited specificity, potentially eliminating senescent cell subpopulations with tissue-repair functions during clearance. Senomorphic agents demonstrate ambiguous therapeutic efficacy, as some clinical trials have failed to confirm significant benefits. Stem cell therapies are limited by the risk of uncontrolled differentiation, which may lead to tumorigenesis or abnormal tissue hyperplasia. Additionally, the safety profile of stem cell transplantation requires rigorous validation to ensure therapeutic reliability. Critically, most clinical trials are hindered by short-term observation periods, leaving long-term safety data insufficiently characterized. Second, standardized and objective biomarkers for clinical evaluation remain underdeveloped, with current antiaging assessments often conflating subjective perceptions with measurable outcomes. While epigenetic clocks are widely utilized to quantify biological aging, these tools remain inadequately standardized, resulting in poor reproducibility and limited cross-study comparability across laboratories. To address these limitations, there is an urgent need to establish consensus-driven, multiomics frameworks, including integrated metabolomic, proteomic, and transcriptomic profiling, to objectively evaluate aging trajectories and therapeutic interventions. Third, the complexity of aging mechanisms and marked individual heterogeneity necessitate the development of personalized therapeutic regimens. Although senolytics exhibit cell type specificity in targeting senescent cells, their clinical translation remains limited by the absence of standardized biomarkers or universally accepted diagnostic criteria to guide precision interventions. Similarly, stem cell-based therapies require customization on the basis of individual genetic profiles; however, current methodologies lack the precision required to achieve spatiotemporal control over cellular differentiation pathways, raising concerns about tumorigenic risks or aberrant tissue remodeling. Fourth, targeting the dynamic, multifactorial nature of aging poses significant therapeutic challenges, particularly owing to transient efficacy and adaptive resistance mechanisms. Aging manifests as a highly complex, temporally evolving process driven by interconnected molecular pathways, rendering monotherapies, such as senolytics, insufficient to achieve comprehensive phenotypic reversal. While combinatorial approaches show promise, the molecular pathways governing such synergies remain poorly characterized. Furthermore, prolonged senolytic administration may trigger compensatory mechanisms, resulting in rebound senescent cell accumulation.

To advance clinical translation, the multidisciplinary integration of cutting-edge technologies is imperative. First, leveraging single-cell multiomics combining transcriptomics, proteomics and epigenomics and AI-driven drug screening platforms will enable the systematic identification of senescence-associated molecular networks and the design of precision therapeutic regimens. Concurrently, establishing consensus-driven multiomics biomarker frameworks is critical for standardizing aging trajectory assessments and tailoring personalized interventions. Second, innovative nanoparticle-based delivery systems should be prioritized to increase the biodistribution and tissue-specific accumulation of therapeutic agents, thereby addressing limitations in current bioavailability. Third, vaccine-based strategies targeting senescence-specific surface markers, such as CD153 or glycoprotein nonmetastatic melanoma protein B (GPNMB), offer a scalable approach for sustained senescent cell clearance. Most importantly, designing reliable clinical trials featuring high-quality, large sample sizes, randomized, double-blind controls, and other rigorous methodologies is crucial for evaluating and developing effective interventions.

## Conclusions and perspectives

### Trends and challenges in the development of longevity drugs

**Fig. 13:**
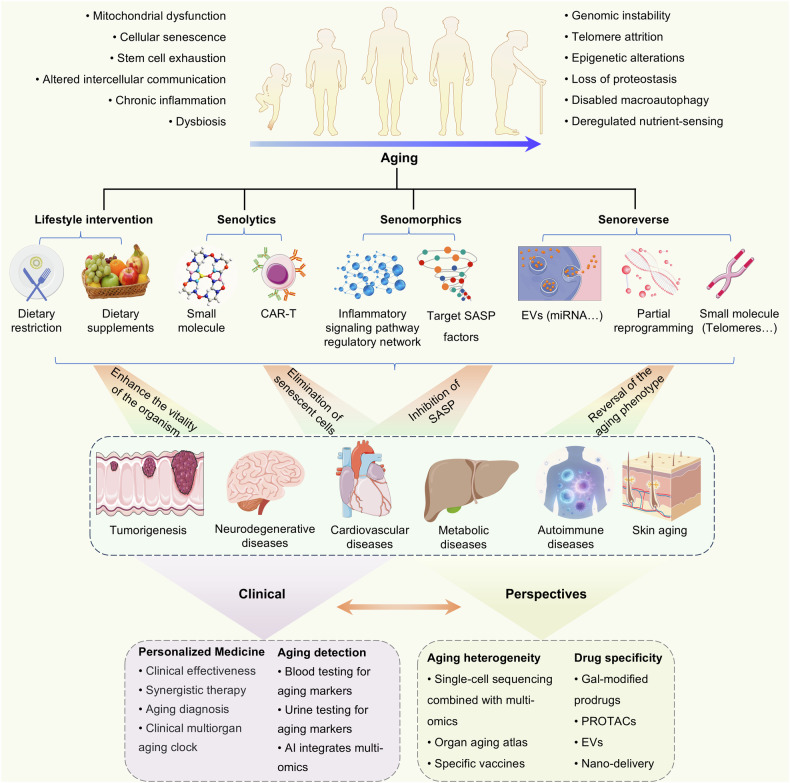
Development and clinical prospects of longevity drugs. The field of antiaging encompasses four distinct therapeutic paradigms: senolytics, senomorphics, senoreverse and lifestyle intervention. These modalities employ diverse molecular mechanisms to address aging and age-related pathologies. However, there are still challenges to the clinical application of these drugs, including drug safety and specificity. The heterogeneity of aging and aging detection markers still need to be addressed in clinical practice

At present, healthy longevity research has exhibited diverse developmental trajectories (Fig. 13). The complexity, heterogeneity, and dynamic nature of cellular senescence highlight the need to develop more precise biomarkers for distinguishing pathological senescent cells from those exerting physiologically beneficial effects during aging. Senolytics and senomorphics represent two emerging therapeutic categories that target age-related diseases. Senolytics can specifically recognize and eliminate senescent cells through the induction of apoptosis. They disrupt antiapoptotic pathways in senescent cells, thereby deactivating their protective mechanisms and reducing pathological accumulation. Preclinical studies have demonstrated therapeutic efficacy across multiple age-related conditions, including osteoarthritis, atherosclerosis, pulmonary fibrosis, and neurodegenerative disorders. However, their clinical translation is limited by potential limitations: 1) impaired tissue repair capacity due to senescent cell clearance, 2) off-target cytotoxicity to nonsenescent cells, and 3) transient inflammatory responses triggered by released cellular contents and inflammatory mediators. These challenges stem from inherent low specificity, off-target effects, and associated toxicities. In contrast, senomorphics exhibit broader therapeutic potential through effective suppression of SASP-related inflammatory factors, thereby attenuating chronic disease progression while preserving the physiological functions of senescent cells. Nevertheless, persistent SASP inhibition may interfere with normal intercellular communication, and therapeutic benefits remain transient owing to SASP reactivation after treatment discontinuation. Furthermore, an incomplete understanding of SASP regulatory networks substantially impedes the development and optimization of novel senomorphics. Emerging evidence suggests that combined strategies integrating SASP inhibition via senomorphics with senescent cell clearance through senolytics may potentiate prolongevity effects.^391^ However, systematic validation of safety profiles and synergistic mechanisms remains imperative before clinical implementation.

To increase the targeting specificity of such therapeutics, senotherapeutic agents are integrated with nanoparticle-based delivery systems to achieve precise intervention in senescent cells. Furthermore, galactose-modified prodrugs demonstrate selective recognition and activity toward senescent cells through β-galactosidase-mediated activation. Specifically, the prodrug undergoes β-galactosidase-catalyzed cleavage in senescent cells with elevated β-galactosidase activity, thereby converting it to its pharmacologically active form to facilitate the selective elimination of senescent cells. This targeting strategy significantly reduced off-target toxicity commonly associated with conventional senolytics. In 2024, Seragon released preclinical data on a new drug, SRN-901, which showed that the oral administration of SRN-901 could prolong the remaining life expectancy of middle-aged mice by 34.4%, exceeding that of rapamycin under the same conditions.^472^ This drug combines NAD^+^ precursors, rapamycin, senolytics, and sirtuin-activating compounds with a pH-responsive gut delivery strategy, resulting in the largest life extension to date for oral aging intervention inhibitors.

The immune clearance strategy of senescent cells, which involves activating or enhancing the ability of the immune system to recognize and kill senescent cells, has become an important research direction. This strategy is based on specific marker proteins on the surface of senescent cells and relies on antigen-receptor recognition to reduce off-target effects and maintain the long-term senescent cell clearance effect. CAR-T cells, antibody-drug conjugates, and senolytic vaccines have been developed to specifically eliminate senescent cells. However, bottlenecks such as immunosenescence and target heterogeneity still need to be overcome. The “senoreverse” strategy has emerged as a transformative solution in the field, utilizing partial epigenetic reprogramming to reverse age-related genetic markers and restore cellular vitality at the molecular level. In addition to reprogramming techniques, emerging rejuvenation modalities include miRNA-loaded exosomes, telomere-activating small molecules, and stem cell transplantation, each of which has the potential to reverse aging phenotypes through distinct mechanisms. Collectively, these innovations represent a comprehensive approach to address the fundamental hallmarks of aging.

### Biomarkers for the detection of aging

Over the past decade, significant progress has been made in the development of novel blood biomarkers of aging. Plasma phosphorylated tau181 (P-tau181) is currently the best blood biomarker for the detection of AD.^473^ Quantitative key indicators of aging, such as IL-23R^474^ and IL-11^475^ in the blood, are used to accurately detect aging. Importantly, large-scale plasma proteomics and machine learning can be leveraged to noninvasively measure organ health and aging in living organisms.^448^ On the basis of a fluorescent probe derived from galactose, a fluorescence platform has been developed for easy longitudinal monitoring of enzymatic activities in biofluids.^476^ The probes are cleaved by β-gal to release fluorophores, which are excreted by the kidneys and can be measured in urine. An in vivo aging detection platform, such as QM-β-gal, has been established to detect senescent cancer cells.^477^ Although these tools hold great promise in trials of longevity interventions, translating them into clinical applications remains challenging. In addition, multiomics techniques such as integrated genomics, transcriptomics, proteomics and metabolomics can elucidate the complex regulatory networks related to aging. These interdisciplinary approaches are expected to reveal novel biomarkers that can predict cellular senescence and the aging trajectory, as well as therapeutic targets for interventions aimed at reversing senescence in tissues. Therefore, multiomics databases must be established to systematically identify and validate biomarkers of aging and to improve the reproducibility of methods and intervention protocols in various studies.

### Personalized medicine and precision approaches to healthy longevity

The escalating global demographic shift toward aging populations has positioned personalized and precision geromedicine as a transformative paradigm for addressing age-associated health burdens. By synergistically leveraging integrative multiomics frameworks, such as epigenomic, proteomic, and metabolomic profiling; AI-driven predictive analytics, and large-scale longitudinal datasets, these approaches enable mechanistic dissection of heterogeneous aging trajectories and targeted modulation of senescence-associated pathways. Critically, such integration facilitates the identification of dynamic biomarkers and the development of personalized therapeutic regimens, including senolytic cocktails and metabolic reprogramming strategies. The integration of personalized medicine and precision interventions signifies a paradigm shift in human healthcare from “passive treatment of diseases” to “active management of aging”. Despite persistent challenges in data integration, ethical controversies, and translational barriers, the synergistic convergence of multiomics technologies, AI, and bioengineering innovations is advancing individualized healthy aging strategies from conceptual frameworks to tangible clinical realities.

Research on aging has entered a new era from phenomenological descriptions to targeted interventions. With the acceleration of the global aging process, aging intervention strategies based on precise mechanisms are not only related to lifespan extension but also a key fulcrum to overcome the predicament of chronic diseases. Traditional treatment strategies focus mostly on a single driving mechanism of aging, such as clearing senescent cells through senolytics, extending telomeres with telomerase activators, or supplementing NAD^+^ precursors to improve mitochondrial function. However, aging is essentially a multidimensional and multisystem coordinated decline process involving various intertwined mechanisms, such as genomic instability, abnormal epigenetic modifications, and protein homeostasis imbalance. Future treatment strategies should be based on a global perspective of the “aging regulatory network”: on the one hand, core regulatory nodes across pathways should be screened through systematic biological analysis; on the other hand, multitarget synergistic intervention technologies, such as combining senolytics with epigenetic modulators to reset cell fate programs while clearing senescent cells, should be developed. The precise detection of aging biomarkers is a prerequisite for personalized treatment. In the future, individual aging maps can be constructed by integrating multidimensional data such as blood metabolomics, gut microbiota metagenomics and epigenetic clocks. On this basis, and combined with artificial intelligence algorithms to predict disease risk, personalized intervention plans are customized.

In summary, future breakthroughs in the antiaging field will rely not only on an in-depth elucidation of aging mechanisms but also on the synergistic advancement of technological innovation and intervention concepts. With the continuous progress of cutting-edge technologies including high-throughput sequencing, single cell analysis and AI, we will be equipped with more powerful tools to probe into aging mechanisms and screen for antiaging targets. In depth investigations into dietary interventions will offer low cost and easily scalable antiaging strategies for the general population, thereby facilitating the popularization of healthy aging concepts. Additionally, the steady advancement of clinical trials for antiaging agents and interventions will accelerate the clinical translation of scientific research findings, ushering in new prospects for the prevention and treatment of age-related diseases. Therefore, exploration of aging mechanisms is not only conducive to clarifying the pathological basis of age-related diseases but also provides new targets and ideas for the development of anti-aging treatment strategies. Through the integrated advancement of multiple disciplines such as molecular biology, medicine, nutrition and engineering, we will establish and refine the aging intervention system, boost the sound development of the anti-aging field, and ultimately realize healthy longevity.
